# Supplementary material for: A high-fat and fructose diet in dogs mirrors insulin resistance and β-cell dysfunction characteristic of impaired glucose tolerance in humans
Source: PLoS One. 2023 Dec 22;18(12):e0296400. doi: 10.1371/journal.pone.0296400 (PMC10745172; doi:10.1371/journal.pone.0296400)
Supplement: S1 File — (PDF) [file pone.0296400.s002.pdf]

Table of Contents

I. PI Contact Record
I d. Requester Biographical Info
II. Protocol Title
Protocol Use Questions
III. Funding Sources
III c. Industry/Commercial Funding
IV. Experimental Procedure Description
V. Scientific Objectives (In Lay Language)
VI. Scientific Benefits (In Lay Language)
XII. Species Use
XII a. Type of Animal Use
XII b. Satellite Housing
XII c. Transportation of Animals
XII d. Species List
Dog--XII d 1. Species Information
Dog--XII d 2. Justification For Animal Use and Choice of Species
Dog--XII d 3. Justification For Number Requested
Dog--Enrichment and/or Exercise
Dog--XII d 6. Non-Surgical Procedures
Dog--XII d 6 i. Non-Surgical Procedure Information
Dog--XII d 7. Surgical Procedures
Dog; Biopsy: Intra-abdominal/laporotomy--XII d 7 i. Surgical Types
Dog; Vascular catheter access--XII d 7 i. Surgical Types
Dog; Placement of catheters into subcutaneous space--XII d 7 i. Surgical Types
Dog; Laparotomy-Vascular Cannulations and Renal Sympathectomy--XII d 7 i. Surgical Types
Dog--XII d 8. Physical Restraint Procedures
Dog; Pavlov’s Harness--Restraint Types
Dog--XII d 9. Hazardous Agents
Dog; triated glucose--XII d 9 i. Hazardous Agents Information

Dog--XII d 10. Drugs/Exogenous Substances (Non Hazardous)
Dog; Pentobarbital--Drug/Substance Information
Dog; Isoflurane--Drug/Substance Information
Dog; Buprenorphine--Drug/Substance Information
Dog; Propofol--Drug/Substance Information
Dog; Meloxicam--Drug/Substance Information
Dog; Insulin--Drug/Substance Information
Dog; indocyanine green--Drug/Substance Information
Dog; normol saline--Drug/Substance Information
Dog; cephalosporin--Drug/Substance Information
Dog; cefpodoxime proxetil--Drug/Substance Information
Dog; heparin-glycerin--Drug/Substance Information
Dog; Somatostatin--Drug/Substance Information
Dog; Glucagon--Drug/Substance Information
Dog; d-Glucose--Drug/Substance Information
Dog; polymeric glucose--Drug/Substance Information
Dog; lidocaine/bupivacaine--Drug/Substance Information
Dog--XII d 11. Euthanasia Methods
Dog; Barbituate Overdose--Euthanasia Method Information
Dog--XII d 13. USDA Pain Categories
Dog--XII d 14. Adverse Consequences
XIII. Personnel List
Cherrington, Alan D--XIII a Personnel Information
Cherrington, Alan D--Personnel Protocol Related Activities
Cherrington, Alan D--Training profile
Williams, Phillip E--XIII a Personnel Information
Williams, Phillip E--Personnel Protocol Related Activities
Williams, Phillip E--Training profile
Moore, Mary C--XIII a Personnel Information
Moore, Mary C--Personnel Protocol Related Activities
Moore, Mary C--Training profile
Smith, Marta--XIII a Personnel Information
Smith, Marta--Personnel Protocol Related Activities
Smith, Marta--Training profile
Farmer, Benjamin N--XIII a Personnel Information

Farmer, Benjamin N--Personnel Protocol Related Activities
Farmer, Benjamin N--Training profile
Farmer, Tiffany--XIII a Personnel Information
Farmer, Tiffany--Personnel Protocol Related Activities
Farmer, Tiffany--Training profile
Scott, Melanie--XIII a Personnel Information
Scott, Melanie--Personnel Protocol Related Activities
Scott, Melanie--Training profile
Edgerton, Dale--XIII a Personnel Information
Edgerton, Dale--Personnel Protocol Related Activities
Edgerton, Dale--Training profile
Hastings, Jon--XIII a Personnel Information
Hastings, Jon--Personnel Protocol Related Activities
Hastings, Jon--Training profile
Adcock, Jamie--XIII a Personnel Information
Adcock, Jamie--Personnel Protocol Related Activities
Adcock, Jamie--Training profile
Kraft, Guillaume--XIII a Personnel Information
Kraft, Guillaume--Personnel Protocol Related Activities
Kraft, Guillaume--Training profile
Gregory, Justin M--XIII a Personnel Information
Gregory, Justin M--Personnel Protocol Related Activities
Gregory, Justin M--Training profile
Fultz, Mary S--XIII a Personnel Information
Fultz, Mary S--Personnel Protocol Related Activities
Fultz, Mary S--Training profile
XIV. Databases Searched
XVII. Hard copy signature option

I. PI Contact Record

Existing contact information will be displayed, based on your login. Information may be added, edited, and saved to the master contact record.

Principal Investigator	Cherrington, Alan D
Protocol Application Number	M/15/147
PI	Cherrington, Alan D
VUNet ID	CHERRIAD
Employee ID	0000758
Primary Role	Principal Investigator
Title	Professor
Organization	Vanderbilt University Medical Center / Molecular Physiology &Bi
Preferred Contact Method (Phone, Email, Cell, etc.)	Phone
Phone	(615) 322-7013
Emergency Phone	(615) 662-4123
Home Phone	
Cell Phone	
Pager	
Fax	(615) 343-0490
Email	alan.cherrington@vanderbilt.edu
Degrees	Ph.D.
Experience and Qualification	More than 50 years working with dogs in research. Involved in the initiation of canine surgery and research program at Vanderbilt University. Internationally known authority on metabolism in the dog. All on-line training up-to-date.

The following grid should display all rooms related to animal research that you use (other than housing locations) specific to this protocol. Please make sure that every room you use for procedures, imaging, restraint, lab work, etc. is included. As you work through the various sections of the protocol form, these rooms will be presented to you for selection based on the purpose that you identify for each room.If a room that you use is not available on the drop down pick list, contact OAWA (343-3586) to have the room added.

Facility/Room No/Purpose
Med Center North/CC1343;;Med Center North/CC2323;;Med Center North/CC2332;;

I d. Requester Biographical Info

Requester	Williams, Phillip E
Degree(s)	B.S.
Organization	Vanderbilt University Medical Center / Surgery - Surgical Resear
Phone	322-3639
Fax	343-1355
Emergency Phone	322-2096
Home Phone	
Cell Phone	615-516-4955
Pager	
Email Id	phil.williams@vanderbilt.edu
Experience and Qualification	Protocol Related Activities: surgery, surgery assistance, anesthesia, euthanasia, husbandry.<br>Mr. Williams is a Research Associate Professor in Surgery and has 40 years experience in an academic research environment. Mr. Williams has 40 years experience with canine, porcine, ovine, caprine and rodent species, specifically with the surgical, anesthetic and experimental procedures relevant to this protocol. During this time he has provided direct supervision of research assistants, fellows, graduate students and medical students in surgical techniques (especially for chronic vascular cannulations), aseptic procedures, animal handling and non surgical procedures, immediate and chronic post- operative and pre-experimental animal care and care of the animal during the experiment.
Preferred Contact Method (Phone, Email, Cell, etc.)	Phone
Primary Role	Co-Investigator
This is the Requester on this Protocol	Yes

II. Protocol Title

The following instructions pertain to the Attachment Section Below

Along with the completed protocol, the IACUC is required to have on file an exact copy of the methods sections and other pertinent information regarding animal use, as submitted in the grant proposal, for all projects. Please attach a copy of the relevant grant sections, including the "Other Project Information Component" (the face page), vertebrate animal section (the five points), and the methods section of the grant application.

For amendments please use the Cover Letter Template and attach with your submission.

Enter title for this protocol
The role of the renal sympathetic nerves in postprandial glucose deposition

Attachments List

File Spec	Description	Created
M_15_147_2_147_1_0001_Blood Draw Tables-Metavention- Renal.doc	Blood collection tables	09/14/2016
M_15_147_2_147_1_0001_Blood Draw Tables-Modified-Metavention.doc	blood draw tables-modified	09/14/2016
M_15_147_2_147_1_0001_Cover letter 5-24-2016-M-15-147.doc	Cover letter 5-24-2016	09/14/2016
M_15_147_2_147_1_0002_Research plan-Renal Denervation-Metaventioon .docx	Research Plan	09/14/2016

Protocol Use Questions

Principal Investigator	Cherrington, Alan D
Protocol Application Number	M/15/147
Will this protocol include field research?	No
Will you be collaborating with an outside institution (other than the Nashville VA)?	No
Will antibodies be	No

produced by an external source?	
Will you produce antibodies in your lab?	No
Will animal tissues or parts be obtained from an external source?	No
Do you have either a primary or joint appointment with the VA?	No

III. Funding Sources

Based on the boxes checked below, additional screens will appear to further identify the fund sources for this proposal. Complete subsequent pages as appropriate.

How will this protocol be funded? Check all applicable boxes	
Agency Funding	
Departmental Funding	
Industry/Commercial Funding	Yes
Fellowship	
Gift	
Other	

III c. Industry/Commercial Funding

Company Name	Metavention LLC.
Private/Commercial Funding Title	The role of renal sympathetic nerves in postprandial glucose deposition
Sponsored Project Fund Number	N/A
Funding Application Due Date	08/01/2015
Does company maintain intellectual property?	YES

Attachments List

File Spec	Description	Created

IV. Experimental Procedure Description

Experimental Procedure Description

Click here to view examples of experimental procedure description.

Provide a *succinct* description of all procedures that will be performed in live animals (from acquisition to the end of your study). ***The IACUC is interested in knowing/understanding WHAT actually happens to each animal: the number of and nature of the procedures, the sequence or order of multiple procedures, and the time involved. Include all recovery or rest periods between manipulations or test periods, and indicate final disposition (e.g., euthanasia, external transfer, retained for additional IACUC approved studies). It is not necessary to describe specifics on non-surgical procedures and surgical procedures in experimental procedure description. This information will come later in the application.***
The purpose of these experiments are to determine if the impairments in glucose metabolism associated with high fat-high carbohydrate dietary intake can be reversed by renal sympathetectomy.<br>Animals will be procured through the Division of animal care from a commercial vendor. The animals will be placed on a high-fat/high-carbohydrate (HFHC) diet and will remain on the diet throughout the course of the experiment. One to two days prior to initiating the HFHC diet and after four and seven weeks of HFHC diet a clinical assessment of glucose metabolism, (oral glucose tolerance test, OGTT), will be performed while the animal is in a pavlov harness to determine the level of glucose intolerance. Glucose will be administered orally and blood samples will be collected over a 4 hour period from a percutaneously placed IV catheter. One week after the 4 week OGTT and after an overnight fast, the animals will undergo a survival surgical procedure in order to place blood sampling catheters such that blood can be collected for the assessment of hepatic and renal metabolism and blood flow cuffs to determine blood flow. In the same surgical setting the animals will be randomly assigned to one of two groups: 1) animals in which bilateral renal

sympathetectomy is performed and 2) those without sympathectomy. Following a two week recovery a third OGTT will be performed. 7-14 days following the third OGTT, two metabolic studies will be performed: a hyperinsulinemic-hypoglycemic study and 7-14 days later a second terminal metabolic study (hyperglycemic-hyperinsulinemic clamp) will be performed. The metabolic studies will be performed following a maximum 23 hr. fast. On the morning of the metabolic studies the catheters (arterial and hepatic and portal venous and flow cuff leads will be removed from the subcutaneous space using a local anesthetic. The animals will be placed in a pavlov harness and two percutaneous IVs will be placed for the administration of the experimental tracers, dyes and hormones. The studies consist of a 100 minute isotopic tracer (3-H glucose) and dye (indocyanine green) equilibration, a 40 minute basal period and a 180 minute study period. Blood will be collected from the surgically placed catheters throughout the experiment. At the conclusion of the first metabolic study the animal will be placed under general anesthesia, the catheters will be placed back into the subcutaneous space under aseptic conditions and the animal will be allowed to recover (procedure requires less than 15 minutes). The animal will then be returned to animal housing and will be fed his daily ration (normal diet). Analgesics and antibiotics will be administered. The animal will be allowed a 7-14 day recovery period and the second metabolic study will be conducted in similar fashion to the first. At the conclusion of the second metabolic study intravenous sodium pentobarbital is administered to induce a deep plane of surgical anesthesia and the animal is transferred from the pavlov harness to a surgical table, a laparotomy is performed and hepatic and skeletal muscle biopsies are collected and the animal is immediately euthanized. This non-survival procedure requires less than 10 minutes.

Attachments List

File Spec	Description	Created

V. Scientific Objectives (In Lay Language)

Specific objectives of this proposed work

How would you explain to a non-scientist the specific objectives of the proposed work? (Please limit your response to 250 words.)
64.5% of U.S. adults are overweight (Body Mass index, BMI, 25) and 33% (BMI 30) are obese. This represents increases of 40 and 114%, respectively, from 1980 to 2011. Severe obesity (BMI 40) prevalence is now 4.7 percent, up from 2.9 percent in 1994. Obesity is associated with a host of co-morbid conditions such as insulin resistance and type II diabetes and cardiovascular pathologies and hypertension. Our understanding of the biological processes associated with the mechanisms behind the development of the co-morbidities is limited. We have previously demonstrated the long term efficacy and safety of the role of hepatic sympathetic nerves and these finding led to a clinical study currently in progress. Recent results from other investigators suggested that renal denervation can improve glucose metabolism in a high fat fed, insulin resistant canine model. We will compare the effect of the renal denervation with our previous data relating to hepatic denervation.

VI. Scientific Benefits (In Lay Language)

Benefits to human or animal health, advancement to knowledge, or societal benefits expected from this proposed animal work.

How would you explain to a non-scientist the way the proposed animal use might benefit human or animal health, the advancement of knowledge, or the good of society?
Obesity is a disease that affects nearly one-third of the adult American population (approximately 60 million). The number of overweight and obese Americans has continued to increase since 1960, a trend that is not slowing down. Each year, obesity causes at least 300,000 excess deaths in the U.S., and healthcare costs of American adults with obesity amount to approximately $100 billion. Understanding the biological processes involved in the development of obesity and the metabolic derangements associated with obesity are critical to developing strategies that can be used to treat the pathologic conditions resulting from diets high in fats and carbohydrates.

XII. Species Use

The following pages will ask questions specific to the species used in this study. After answering all of the questions pertaining to a species, you will be directed back to the Species List page (XII d 1.) to add additional species as needed.

Confirm that you will be using animals on this protocol.	YES

XII a. Type of Animal Use

Identify all types of animal use for this protocol. Your choices here and on subsequent pages will determine the correct USDA pain category. Click here to view Vanderbilt's guidelines on breeding.

Which of the following describes the type of animal use proposed in this application? (check all that apply)	
Research	Yes
Teaching	
Other (Specify)	

XII b. Satellite Housing

Approved satellite rooms will be displayed in the "Location" pick list. If the room is not on the list, you must apply to have a new satellite room approved. The approval process could significantly delay protocol approval. Click here to view Vanderbilt's SOP on satellite animal housing facilities.

Will animals be housed outside the DAC managed animal housing facilities for more than 12 hours?	NO
If yes, please provide following information:	
Location	
Scientific Justification	

XII c. Transportation of Animals

Transportation of animals outside DAC managed facilities must follow guidelines set by the IACUC. Please click Procedures for the Transport of Animals at Vanderbilt University .

Will you be moving animals through public access areas?	NO
If "Yes", which transportation route(s) described in the Approved Transportation Routes do you intend to use (list all that apply).	
If you intend to use a route that is not described in the SOP, please describe your route:	

XII d. Species List

Indicate which species you propose to use for this study.

Hint: If you have already entered the information for one species and are adding other species to the protocol, click the "select" button for the original species and use the *Copy Species* button if you are duplicating any procedures.

Species	# Requested
Dog	13

XII d 1. Species Information

Choose proposed species from the pick list. If species is not available, contact the OAWA/eSirius helpline at 343-3586. You will only be able to request breeding cage cards if you check "Breeding" as one of the types of animal use below.

Species	Dog

Species Activities

Will drugs be administered on this Species?	Yes
Will animals be euthanized?	Yes
If not, what will be done to animals after the experiment or project is completed?	
Will non-surgical procedures (i.e. blood collection, tissue collection, etc.) be performed?	Yes
Will surgeries be performed?	Yes
Will animals be restrained?	Yes
Will hazardous agents be used?	Yes
Breeding?	NO

XII d 2. Justification For Animal Use and Choice of Species

Species Name	Dog

Please explain the rationale for the use of animals on this protocol and the species you propose to use.
This protocol will address whether or not renal sympathetic denervation has an effect on the development of insulin resistance induced by a high fat high, high carbohydrate diet. The questions proposed involve metabolic processes that are regulated by multiple organ systems of the body (for example, liver and brain have integrated metabolic regulation of glucose production and utilization) and these complex metabolic relationships cannot be studied in single cell, single organ experimental settings. In-silico or computer modeling methodologies are inadequate due to complexity of variables therefore, an animal model is required for the outlined experiments. Our laboratory has extensive experience in the use of the canine model to address questions relating to the control of glycemic states and over the years we have

developed a wide array of surgical and pharmacologic tools that enable us to break the feedback loops that are associated with glycemic control in the whole animal. We can induce obesity in canines by feeding a high fat, high carbohydrate diet. These animals demonstrated a 15-20% weight gain and glucose intolenance. Due to our ability to intervene surgically, the dog can be used for experiments that simply cannot be carried out in the human and the animal's size permit time-course experiments to be conducted that cannot be conducted in smaller animals due to need to address blood sample size and analytical requirements. Finally, the physiological process of glycogenolysis and gluconeogenesis of the canine closely resembles human physiology and indeed much of what we have learned in the canine has been extrapolated to the human

XII d 3. Justification For Number Requested

All animals generated in the proposed protocol must be reported in this section. This includes animals used for breeding, experiments and unused offspring. Using the specifics of your experimental plan, demonstrate how the numbers of animals required to achieve your scientific objectives for this project were calculated. Briefly detail the experimental design so that the reviewers can ascertain how the animals are used. Include details of number of animals per group, control groups, treatment groups, pilot studies, and potential experimental failure. Provide statistical justification for the number of animals in experimental groups as well as justification for the total number of animals requested.
If breeding is part of the proposed protocol, make sure include breeding in a separate paragraph. Indicate the number of breeders to be mated to generate the animals needed in the experiments. Also, describe the mating scheme (1 male per 1 female, 1 male for 2 females, etc.). Indicate the proposed number of litters per female and the expected number of pups per litter.

(Note: Approved numbers may not be exceeded without an amendment.)

Species Name	Dog

Total number of requested animals for a 3-yr period
13
What is your justification for number of animals requested?
12 animals are required to complete the studies in this proposal.The protocol will utilize two groups of animals that are placed on a high fat/high carbohydrate diet. Group 1(N=6) will undergo surgery for the vascular catheter placement and renal sympathectomy. The second(control)group (n=6)involves

only the surgery for the vascular catheter placement.<br><br>Power calculations were performed based on anticipated variances in the groups and expected observations. The n required to provide the power to detect the expected outcome was calculated with SigmaStat (Systat Software, Point Richmond, CA) with 90% confidence, with a type I error of 0.05. The variance was assumed based on responses in previous groups of dogs from our laboratory. The N=6 is based on the expected variance (1.5 mg/kg/min) of whole body glucose uptake. One additional animal is requested to compensate for any catheter or flow cuff failures or unforseen experimental problems. Thus a total of 13 animals are requested.

Attachments List

File Spec	Description	Created

Enrichment and/or Exercise

The Animal Welfare Act requires facilities to provide exercise for dogs and programs to promote the psychological well-being of non-human primates, while the U.S. Public Health Service Guide to the Care and Use of Laboratory Animals encourages "enriching the environment as appropriate to the species....".

Species Name	Dog
How will animals be housed?	Singly
If housed in pairs/groups with a proposed period of separation that exceeds 12 hours in every 24 hour period, provide the scientific justification for the period of separation (single housing).	Single housing is needed for the provision of consistent daily nutrition and daily intake assessment and metabolic equilibrium. A stable and consistent metabolic profile is a critical requirement of the proposed studies. Metabolic regularity can be affected by many environmental and procedural factors including physical activity, dietary intake, interaction with personnel and study procedures. The animals are placed on a high fat-high carbohydrate diet fed ad lib and daily dietary intake of each animal must be assessed daily. To ensure daily metabolic consistency and assess dietary intake the animals are housed singly and provided with 4-6 hours daily socialization with other animals. During the time the animals are housed there is significant human social interaction. The daily activities include assessments of clinical condition and behavior, provision of daily diets and observations of social interaction with other animals. In addition the animal caretaker s daily activities include cage enclosure

	maintenance and general facility/room maintenance. Thus there is significant interaction and observation of the animals throughout their tenure. Permitting the animals to socialize during daily activities, followed by the afternoon and evening isolation for dietary intake and assessment of intake ensures consistent and routine metabolic patterns and ensures animal welfare during times in which animals cannot be under observation.
Will animals participate in VUMC Canine Excercise Plan?	Yes
If not, please provide scientific justification for exemption from VUMC Canine Exercise Plan:	
Indicate whether the following enrichment items may be used:	
Structural EE (i.e. dog beds, etc...):	Yes
Manipulanda (i.e. chew toys - rubber and/or plastic):	Yes
Food treats/Foraging:	No
Training (i.e. sit, stay, come - positive reinforcement/clicker):	Yes
Positive Human Contact:	Yes
Please provide scientific justification for all enrichment restrictions from above:	The provision of food other than normal daily rations has the potential to result in appropriate fasting times. Supplementary treats or food stuffs could adversely affect the outcome of the study for several reasons. It is very important that the dogs remain on a constant diet throughout the pre-study &study period. Therefore additional food items could disrupt the constant diet. Further, additional food items could affect the dogs appetite so that they will not consume the study diet, thereby affecting the constant diet.

XII d 6. Non-Surgical Procedures

Indicate the non-surgical procedures you will conduct. Your choices of procedures will determine the USDA pain level for each species requested. Click here to view Vanderbilt's guidelines on detecting pain.

Species Name	Dog

To find information regarding different adjuvants, administration routes, and pain, click here

Antibody Production   Environmental Modification   Diet Manipulation   Diabetes Studies   Irradiation

Procedure Name
Blood/fluid/tissue Collection (ante-mortem)
Diet Manipulation, Including Food/water Regulation

Attachments List

File Spec	Description	Created

XII d 6 i. Non-Surgical Procedure Information

Species Name	Dog

Details about the procedure:

**Blood/fluid/tissue Collection (ante-mortem)**	
Specify the fluid as well as the site and method of fluid collection. If collection involves single cage housing or housing in non-standard cage, Environmental Modification section section must be completed.	1)Oral Glucose Tolerance Test: Blood collected from a percutaneously placed venous catheter or a surgically placed femoral vein catheter.<br>2)Metabolic Study 1: Blood collected from indwelling catheters in the femoral artery.<br>3)Metabolic Study 2: Blood collected from indwelling catheters in the hepatic vein, portal vein and femoral artery.<br>4)blood for clincal assessment, peripheral venopuncture
Indicate the amount of fluid collected during one procedure, frequency	1) OGTT up to 97 ml collected over 4 hr. Up to three test will be performed with a minimum of one week between the assessments.<br>Metabolic Study 1:up to 150 ml collected over 6 hr. (at 5-15

and interval of collections.	minute intervals)<br>3)Metabolic Study 2: Up to 276 ml collected over 6 hr (at 5 and 15 minute intervals). The animals are euthanized at the conclusion of the second metabolic study. The blood volume of canines is 8% of body weight and we will collect no more than 25% of the animals blood volume over the course of an experiment (6 Hr.). Normal saline will be replaced at a 2:1 ratio: Our animals are generally 20kg. A general example of blood collection protocol is presented in Tables 1 and 2 of attached document. Our experience has indicated that these volumes of blood can be withdrawn safely and without metabolic consequences within the experimental timeframes. The animals do not exhibit any adverse metabolic conditions such as increased heart rate, increased respiratory rate, or increased circulating concentrations of epinephrine norepinephrine or cortisol which are monitored in all experiments. Additionally, these animals are monitored continuously during the course of the blood collections. Any demonstrated stress (dyspnea, weakness,) in addition to metabolic conditions mentioned above will result in termination of the procedure or early euthanasia of the animal.<br>3) up to 20ml (collected for pre-surgical and pre experimental assessments on five occasions.
Will the animal be anesthetized?	no
**Diet Manipulation, Including Food/water Regulation**	
Describe the diet manipulation.	1) Food will be withheld for 23 hrs prior to all experiments (OGTT and metabolic experiments). Water will be provided ad lib. 23 hour fasting is required for all experiments to ensure that complete digestion of the meal has occurred and no glucose or substrates are entering the portal venous system. In all experiments we monitor inflowing substrates (glucose, lactate, amino acids)into the portal venous system to verify that the absorptive/digestive period is complete. Our data and that of others has shown that the post-prandial absorptive period is complete by 18-24 hr. (Davis MA, Williams PE, and Cherrington AD. Effect of a mixed meal on hepatic lactate and gluconeogenic precursor metabolism in dogs. Am J Physiol 247:E362-E369, 1984). In addition, this

	length of fast (overnight) ensures a consistent metabolic presentation between individual animals.<br><br>2)Animals will be fed a high-fat high carbohydrate diet, (45 kcal%) containing 25 kcal% carbohydrate ad lib. The animals will be fed this diet over a period of up to 8 weeks. Water will be provided ad lib. The diet will be provided by laboratory personnel and recorded daily.
How long will the animal be maintained in this particular nutritional state?	1)up to 8 hr. During the experiment the animals will receive intravenous glucose to maintain glycemia.<br>2) up to 8 weeks
Will free access to water be restricted? If yes, please answer the questions below.	no
Please state the duration of water regulation.	N/A
Do you follow VU SOP for fluid regulation?	N/A
If not, please describe deviation from SOP.	N/A

Select the room where this procedure will be performed. If the room is not on the displayed list, you will need to go back to the PI Contact Information Page and add the room there and then return to select it for this procedure.

Select Room(s)	Med Center North/CC1343;;Med Center North/CC2323;;Med Center North/CC2332;;

XII d 7. Surgical Procedures

Click here to view Vanderbilt's policy on surgical techniques.

Species Name	Dog

Surgery	Survival?	MMS?	MMS Rationale
Biopsy: Intra-abdominal/laporotomy	Non-survival		
Vascular catheter access	Survival		
Placement of catheters into subcutaneous space	Survival		
	Survival		

Laparotomy-Vascular Cannulations and Renal Sympathectomy				

Protocol Surgery Type - Multiple Major Survival Surgeries

Click here to view Vanderbilt's SOP on Multiple Major Survival Surgeries and Relevant Recordkeping in all Species.

Will Multiple Major Survival Surgeries be performed?	NO
If yes, please provide scientific justification and expected number of major surgeries	

XII d 7 i. Surgical Types

Describe the planned surgery. For assistance in completing this form, contact DAC veterinarian at 936-1820. All animals undergoing survival surgical procedures must have documented observations for a minimum of once daily for three days.

Species Name	Dog

Choose Surgery type from list. If desired surgery is not on list, please enter in the **Other Surgery** field.

Type of surgery to be performed	Biopsy: Intra-abdominal/laporotomy
Is this a **Survival** or **Non-survival** Type of Surgery	Non-survival
Will pre-operative care involve the withholding of food?	No
If yes, for how many hours?	29.00
If >12 hours, please justify:	Food will be withheld for 23 hrs prior to the experiment. The duration of the experiments are generally 6 hr., thus the fasting period prior at the end of the experiment and collection of tissues is a maximum of 29 hr. Nutirional support is provided with intravenous glucose is throughout the experiment. 23 hour fasting is required for these experiments to ensure that complete digestion of the meal has occurred and no glucose or substrates are entering the portal

	venous system. In all experiments we monitor inflowing substrates (glucose, lactate, amino acids)into the portal venous system to verify that the absorptive/digestive period is complete. Our data and that of others has shown that the post-prandial absorptive period is complete by 18-24 hr. (Davis MA, Williams PE, and Cherrington AD. Effect of a mixed meal on hepatic lactate and gluconeogenic precursor metabolism in dogs. Am J Physiol 247:E362-E369, 1984). In addition, this length of fast (overnight) ensures a consistent presentation of metabolic status between animals.
List anesthetic drugs used for this specific surgery (Please do not include dosages).	sodium pentobarbital
Will analgesics be administered pre-operatively?	No
If pre-operative analgesics are not administered, please provide a justification here.	non-survival
Will aseptic technique be followed as described in SOPs for Non-Rodent or Rodent surgery?	No
If No, describe deviation from SOP	non-survival
Describe how the animals will be monitored during surgery including anesthetic depth. If paralysis is induced, please describe monitoring during paralysis either via heart rate &blood pressure or EEG.	animals will be monitored for lack of reflex during the procedure. No surgical procedures will be performed until this is an absence of response to toe pinch and eyelid reflex.

Describe the surgical procedure. Please describe post-op care for NMB. Please describe that you have verified that the NMB drug has worn off before anesthesia is discontinued. Please be sure animal has been anesthetized at least 20 minutes or surgical procedure performed before drug is administered.	Pentobarbital sodium is administered IV. A midline (6 in.) laparotomy is performed. Access to the liver will be gained by blunt dissection. Three biopsies (2-4 gm) will be taken from the left and right lateral and a medial lobe of the liver. The biopsies will be snap frozen in liquid nitrogen and stored. A 2 in. incision will be made on the medial thigh, and the sartorius and gracilis muscles will be approached by blunt dissection. 500 mg samples will be taken from each muscle with the scalpel and frozen in liquid nitrogen. Once the biopsies are collected the animal is immediatley euthanized.
Duration of surgery	10 minutes
Will drugs such as antibiotics, fluids, etc. be given post-procedurally (If so list in Drugs section XII d 10)?	NO
Will analgesics be used post-operatively?	No
If analgesics are administered post-operatively, please provide the name(s) and duration of administration of analgesic agents. Do not include drug dosages in this section. If analgesics are not administered post-operatively, please provide justification here.	

Describe the clinical signs that would result in the administration of analgesia	not applicable
Wound Closure (state filament type, number of layers, and absorbable v. nonabsorbable)	not applicable
When will Sutures be Removed?	
Describe post-operative care procedures including observation frequency and expected duration of observation period	not applicable
How long will animal be maintained following surgery?	not applicable
Will neuro-muscular blocking agents (paralytics) be used? If yes, be sure to include on drugs page (XII d 10)	No

Use locations

Select the room where this procedure will be performed. If the room is not on the displayed list, you will need to go back to the PI Contact Information Page and add the room there and then return to select it for this procedure.

Select Room(s)	Med Center North/CC1343;;Med Center North/CC2323;;

XII d 7 i. Surgical Types

Describe the planned surgery. For assistance in completing this form, contact DAC veterinarian at 936-1820. All animals undergoing survival surgical procedures must have documented observations for a minimum of once daily for three days.

Species Name	Dog

Choose Surgery type from list. If desired surgery is not on list, please enter in the **Other Surgery** field.

Other Surgery	Vascular catheter access
Is this a **Survival** or **Non-survival** Type of Surgery	Survival
Will pre-operative care involve the withholding of food?	Yes
If yes, for how many hours?	23.00
If >12 hours, please justify:	The animals are fasted for 23 hr. prior to experiment. Catheter access occurs immediately prior to metabolic experiment. 23 hour fasting is required for these experiments to ensure that complete digestion of the meal has occurred and no glucose or substrates are entering the portal venous system. In all experiments we monitor inflowing substrates (glucose, lactate, amino acids)into the portal venous system to verify that the absorptive/digestive period is complete. Our data and that of others has shown that the post-prandial absorptive period is complete by 18-24 hr. (Davis MA, Williams PE, and Cherrington AD. Effect of a mixed meal on hepatic lactate and gluconeogenic precursor metabolism in dogs. Am J Physiol 247:E362-E369, 1984). In addition, this length of fast (overnight) ensures a consistent presentation of metabolic status between animals.
List anesthetic drugs used for this specific surgery (Please do not include dosages).	lidocaine/bupivacaine
Will analgesics be administered pre-operatively?	Yes
If pre-operative analgesics are not	

administered, please provide a justification here.	
Will aseptic technique be followed as described in SOPs for Non-Rodent or Rodent surgery?	Yes
If No, describe deviation from SOP	
Describe how the animals will be monitored during surgery including anesthetic depth. If paralysis is induced, please describe monitoring during paralysis either via heart rate &blood pressure or EEG.	Heart rate, respiratory rate, and response to external stimuli will be monitored continuously. The procedure required less that 15 minutes.
Describe the surgical procedure. Please describe post-op care for NMB. Please describe that you have verified that the NMB drug has worn off before anesthesia is discontinued. Please be sure animal has been anesthetized at least 20 minutes or surgical procedure performed before drug is administered.	Following the administration of the local anesthetic a small 1cm skin incision is performed over the subcutaneous catheter and flow cuff sites on the abdomen. Blunt dissection is performed and the catheters and Doppler flow leads are removed from their subcutaneous pockets.
Duration of surgery	less tha 15 minutes
	YES

Will drugs such as antibiotics, fluids, etc. be given post-procedurally (If so list in Drugs section XII d 10)?	
Will analgesics be used post-operatively?	Yes
If analgesics are administered post-operatively, please provide the name(s) and duration of administration of analgesic agents. Do not include drug dosages in this section. If analgesics are not administered post-operatively, please provide justification here.	additional lidocaine/bupivacaine will be administered if clinical signs are apparent.
Describe the clinical signs that would result in the administration of analgesia	Clinical manifestations that would indicate the need for additional analgesia are; growl or apprehension when approached and guarding of surgical sites.
Wound Closure (state filament type, number of layers, and absorbable v. nonabsorbable)	The skin incision is closed in a single interrupted fashion using a non-wicking monofilament non-absorbable suture.
When will Sutures be Removed?	N/A
Describe post-operative care procedures including observation frequency and expected duration of observation period	Wound site will be obeserved continuusly throughout the experiment.

How long will animal be maintained following surgery?	6 hr
Will neuro-muscular blocking agents (paralytics) be used? If yes, be sure to include on drugs page (XII d 10)	No

Use locations

Select the room where this procedure will be performed. If the room is not on the displayed list, you will need to go back to the PI Contact Information Page and add the room there and then return to select it for this procedure.

Select Room(s)	Med Center North/CC1343;;Med Center North/CC2323;;Med Center North/CC2332;;

XII d 7 i. Surgical Types

Describe the planned surgery. For assistance in completing this form, contact DAC veterinarian at 936-1820. All animals undergoing survival surgical procedures must have documented observations for a minimum of once daily for three days.

Species Name	Dog

Choose Surgery type from list. If desired surgery is not on list, please enter in the **Other Surgery** field.

Other Surgery	Placement of catheters into subcutaneous space
Is this a **Survival** or **Non-survival** Type of Surgery	Survival
Will pre-operative care involve the withholding of food?	YES
If yes, for how many hours?	30.00
If >12 hours, please justify:	The procedure is performed immediately following the experiment. The animals are fasted for 23 hr. prior to the experiment and the experiment will last a maximum of 7 hr., thus 30 hr of fast will occur. During the experiment the animals will receive intravenous glucose for glycemic maintenance during hyperinsulinemia. The minor surgical

	procedure last approximately 15 minutes. The animal is anesthetically recovered and returned to the housing area. He is fed his daily ration at that time.
List anesthetic drugs used for this specific surgery (Please do not include dosages).	propofol (induction), isoflurane with O2 (maintenance)
Will analgesics be administered pre-operatively?	YES
If pre-operative analgesics are not administered, please provide a justification here.	
Will aseptic technique be followed as described in SOPs for Non-Rodent or Rodent surgery?	YES
If No, describe deviation from SOP	
Describe how the animals will be monitored during surgery including anesthetic depth. If paralysis is induced, please describe monitoring during paralysis either via heart rate &blood pressure or EEG.	Heart rate, respiratory rate, SPO2, body temperature will be monitored continuously and recorded at 15 min intervals. Response to external stimuli, withdrawal and palpebral reflex will be monitored continuously during the procedures
Describe the surgical procedure. Please describe post-op care for NMB. Please describe that you have verified that the	Placement of catheters into subcutaneous space following the experiment. At the completion of each survival experiment the catheters and flow cuff leads will be returned to the original subcutaneous pocket which was made at the time of surgical preparation (immediately adjacent to the midline laparotomy). This procedure will be performed at the end of the metabolic study. The animals will be placed under general

NMB drug has worn off before anesthesia is discontinued. Please be sure animal has been anesthetized at least 20 minutes or surgical procedure performed before drug is administered.	anesthesia for this procedure; the catheters will be washed and aseptically cleansed with betadine/alcohol (3 times). The catheters will be filled with a sterile mixture of heparin and glycerin (1000U/ml in 1 to 1 ratio), and their free ends will be knotted and placed in a subcutaneous pocket at the site of catheter exposure. Wound closure will be performed in two layer fashion (SQ and skin).
Duration of surgery	0.25 hr
Will drugs such as antibiotics, fluids, etc. be given post-procedurally (If so list in Drugs section XII d 10)?	YES
Will analgesics be used post-operatively?	YES
If analgesics are administered post-operatively, please provide the name(s) and duration of administration of analgesic agents. Do not include drug dosages in this section. If analgesics are not administered post-operatively, please provide justification here.	Buprenex and meloxicam are provided pre operatively providing 24 hr. analgesic coverage. Additional analgesia is provided if required.
Describe the clinical signs that would result in the administration of analgesia	Clinical manifestations that would indicate the need for additional analgesia are; unusual quietness, unwillingness to move, lack of alertness, whimpering or howling, increased respiration, growl or apprehension when approached and guarding of surgical sites.
Wound Closure (state filament type,	The surgical site will be closed in two layer fashion with single interrupted sutures using absorbable suture for

number of layers, and absorbable v. nonabsorbable)	subcutaneous tissues and the skin is closed in single horizontal mattress fashion using a non-wicking monofilament suture
When will Sutures be Removed?	10-14 days
Describe post-operative care procedures including observation frequency and expected duration of observation period	Post-operative care and observation is provided daily for 3 days. The care involves the examination of the wound sites, assessment vital signs assessment of dietary intake, administration of post-operative medications
How long will animal be maintained following surgery?	up to 14 days
Will neuro-muscular blocking agents (paralytics) be used? If yes, be sure to include on drugs page (XII d 10)	NO

Use locations

Select the room where this procedure will be performed. If the room is not on the displayed list, you will need to go back to the PI Contact Information Page and add the room there and then return to select it for this procedure.

Select Room(s)	Med Center North/CC1343;;Med Center North/CC2323;;Med Center North/CC2332;;

XII d 7 i. Surgical Types

Describe the planned surgery. For assistance in completing this form, contact DAC veterinarian at 936-1820. All animals undergoing survival surgical procedures must have documented observations for a minimum of once daily for three days.

Species Name	Dog

Choose Surgery type from list. If desired surgery is not on list, please enter in the **Other Surgery** field.

Other Surgery	Laparotomy-Vascular Cannulations and Renal Sympathectomy
Is this a **Survival** or **Non-survival** Type of Surgery	Survival
Will pre-operative care involve the withholding of food?	Yes
If yes, for how many hours?	12.00
If >12 hours, please justify:	
List anesthetic drugs used for this specific surgery (Please do not include dosages).	propofol (induction), isoflurane with O2 (maintenance)
Will analgesics be administered pre-operatively?	Yes
If pre-operative analgesics are not administered, please provide a justification here.	
Will aseptic technique be followed as described in SOPs for Non-Rodent or Rodent surgery?	Yes
If No, describe deviation from SOP	
Describe how the animals will be monitored during surgery including anesthetic depth. If paralysis is induced, please describe monitoring during paralysis either via heart	Heart rate, respiratory rate, end tidal CO2, body temperature will be monitored continuously and recorded at 15 min intervals.

rate &blood pressure or EEG.	
Describe the surgical procedure. Please describe post-op care for NMB. Please describe that you have verified that the NMB drug has worn off before anesthesia is discontinued. Please be sure animal has been anesthetized at least 20 minutes or surgical procedure performed before drug is administered.	All animals in will have a laparotomy for the purpose of vascular cannulations and placement of flow cuffs in order to measure hepatic and renal blood flow. One group of animals will have a bilateral renal sympathectomy performed in the same surgical setting in addition to vascular cannulations and flow cuff placements. The vascular cannulations include; hepatic and portal and renal venous cannulations and placement of flow cuffs, catheterizations for intraportal infusions and inferior vena cava and femoral artery cannulation. Silastic tubing (Technical Products Inc, Decatur Ga.) will be used as catheter material and will be placed in arteries and veins, filled with heparinized saline glycerin/heparin(500 U/ml) and their free ends will be exteriorized through the incision, knotted and placed in a subcutaneous pocket. Blood flow will be assessed with continuous doppler methodology and cuffs (Transonics Systems, Ithaca, N.Y.) are placed around the portal vein and hepatic artery. Cuff leads are exteriorized and placed in the same fashion as the catheters. Hemostasis is controlled by electrocaughtry and/or vessel ligation with suture material. Incisions and operative sites are closed in 3 layer fashion; muscle/peritoneum, subcutaneous tissues and the skin. The specific surgical procedures are detailed below:<br>Hepatic and portal venous cannulations and placement of flow cuffs - following a midline laparotomy exposure to the left common hepatic vein and left branch of the portal vein is achieved by retracting the left lateral lobe caudally and the left medial lobe anteriorly. Purse string sutures are placed on the respective vessels and a 12 ga. trocar is inserted into the vessels. The stylet of the trocar is removed and 0.040 in. ID silastic catheter is advanced into the vessels. The tip of the hepatic vein catheter is positioned 1.5 cm in the left common hepatic vein and the tip of the portal vein catheter is positioned 5 cm retrograde into the portal vein. The catheters are secured with their respective pursestring suture and two additional single interrupted sutures. Blood flow is assessed by cuffs placed on the common hepatic artery (4.0 mm) and portal vein (6.0-8.0 mm), distal to the gastroduodenal vein which will be ligated.<br>Renal Venous cannulation and placement of flow cuff :

Exposure to the left renal vein is accomplished by medial retraction of the intestinal and splenic beds such that exposure to the left renal vein at the convergence to the inferior vena cava at the point of is gained. A purse string suture is placed on the renal vein and a venotomy is made on the left renal vein 1 cm from the inferior vena cava using an 12 G needle. A silastic catheter (0.040 in. ID) is inserted and advanced 3.0 cm into the vein and the purse string suture is closed. The catheter is sutured to the inferior vena cava. A 3.0 mm flow cuff is placed around the left renal artery for the assessment of blood flow.

Catheterizations for inferior vena cava infusion: Exposure to the inferior vena cava is accomplished by medial retraction of the intestinal and splenic beds such that exposure to the inferior vena cava is gained. An venotomy is made using an 12 G needle at the level of the renal vein anastomosis with the inferior vena cava and a silastic catheter (0.040 in. ID) is inserted and advanced 4.0 cm into the vein. A purse string suture is placed and closed. The catheter is sutured to the inferior vena cava.

Catheterizations for intraportal infusions: Two silastic catheters (0.030 in. ID, Technical Products Inc., Decatur, Ga.) are placed in a branch of the jejunal vein and a branch of the splenic vein such that their tips are advanced beyond the first coalescing vessel. This permits adequate mixing of the infusions with blood prior to reaching the liver

Catheterization of the femoral artery: The left or right femoral artery is catheterized and the tip of the catheter is advanced into the distal aorta. Blood from this site serves as the representative arterial blood for all organ and tissue systems. An incision is made over the femoral artery approximately 2 cm distal to the left lateral circumflex artery. The tissue is dissected to gain exposure of the artery and the distal end of the artery is ligated. An arteriotomy is performed and a silastic catheter is introduced into the artery and advanced to the level of the distal aorta (approximately 16 cm). The catheter is filled with heparinized saline (200 U/ml) and its free end is knotted and placed in a subcutaneous pocket.
since these involve

Bilateral Renal Sympathectomy: Access to the renal vascular bed is obtained by retraction of the omentum and intestinal

	bed laterally. The renal vascular bed (left and right renals arteries and veins) is carefully dissected from the connective tissues and renal fat pedicle and the distal aorta and left and right renal arteries are exposed and the nerves surrounding each renal artery are stripped from the vessel and resected from the point of take-off from the aorta to 1cm distal to the renal hilus. The surgical denervations result in a 98% decrease in the total norepinephrine content of each kidney.
Duration of surgery	1.5 hr.
Will drugs such as antibiotics, fluids, etc. be given post-procedurally (If so list in Drugs section XII d 10)?	YES
Will analgesics be used post-operatively?	Yes
If analgesics are administered post-operatively, please provide the name(s) and duration of administration of analgesic agents. Do not include drug dosages in this section. If analgesics are not administered post-operatively, please provide justification here.	Buprenex is provided pre and 8 hr. post operatively. In addition, meloxicam is provided pre and 24 hr. postoperatively providing analgesic coverage for 48 hr. Additional analgesia is provided if needed.
Describe the clinical signs that would result in the administration of analgesia	Clinical manifestations that would indicate the need for additional analgesia are; unusual quietness, unwillingness to move, lack of alertness, whimpering or howling, increased respiration, growl or apprehension when approached and guarding of surgical sites.
Wound Closure (state filament type, number of layers, and absorbable v.	Incisions and operative sites are closed in 3 layer fashion; muscle/peritoneum, closed in a single interrupted fashion using an absorbable suture; subcutaneous tissues closed in continuous lock stitch fashion using absorbable suture and

nonabsorbable)	the skin is closed in single horizontal mattress fashion using a monofilament nylon nonabsorbable suture.
When will Sutures be Removed?	10-14 days
Describe post-operative care procedures including observation frequency and expected duration of observation period	Animals are monitored continuously in the immediate postoperative period until as demonstration of the ability to maintain sternal recumbency and to exhibit purposeful movement.<br>Daily post-operative care and observation is provided for 3 days. Analgesic coverage is provided for 48 hr. The care involves the examination of the wound sites, assessment vital signs and body temp., assessment of dietary intake and urinary and gastrointestinal function, administration of post-operative medications
How long will animal be maintained following surgery?	up to 8 weeks
Will neuro-muscular blocking agents (paralytics) be used? If yes, be sure to include on drugs page (XII d 10)	No

Use locations

Select the room where this procedure will be performed. If the room is not on the displayed list, you will need to go back to the PI Contact Information Page and add the room there and then return to select it for this procedure.

Select Room(s)	Med Center North/CC2332;;

XII d 8. Physical Restraint Procedures

Click here to view Vanderbilt's guidelines on restraint.

Species Name		Dog
Restraint Type	Duration	Rationale
Pavlov's Harness	Up To 6 Hrs	These experiments address the effects of hormones and substrates on metabolic physiolgy. The studies require constant infusion of these compounds and samples are collected frequently (up to five minute intervals). There are multiple sampling sites and intravenous and arterial cannulas that

		require attention and care. Therefore maintaining the animals in a confined but comfortable postion is necessary.

Restraint Types

Describe how you will minimize potential distress from restraint in the rationale section below

Species Name	Dog

Choose restraint from list. If desired restraint is not on list, please enter in **Other Restraint** field. Only list methods of physical restraint (not chemical via anesthesia) longer than 10 minutes.

Restraint Type	Pavlov’s Harness
How is animal acclimated to the restraint device	One of our primary considerations in the choice of breeders and suppliers is the socialization and relaxed demeanor of each animal. We insist that the animals be well conditioned and well socialized prior to assignment to protocol. This is an absolute necessity for these conscious studies. Animals that do exhibit fear, distress or anxiety exhibit physiologically high cortisol and catecholamine levels which are detrimental to the metabolic observations we are concerned with and therefore are not appropriate for the studies. We observe these animals at least on a daily basis for 5 days or more prior to the surgical procedure to ensure that the animals can be handled by personnel and do not exhibit a demeanor or disposition of fear, anxiety, excessive timidness, stress or excessive activity. Animals exhibiting normal calm activities are not acclimated to the harness prior to an experiment. Animals that exhibit a questionable disposition or fear are acclimated to the harness on at least one (and up to three) occasion(s) to determine if they can acclimate and relax (comfortable, resting quietly and generally relaxed) in that setting. If the animals are not comfortable or exhibit an unwillingness to relax they are reassigned to protocols not involving conscious studies. While in the Pavlov harness animals are in constant visual contact with lab personnel and a minimum of one adult human in the room with the dog. There is extensive physical contact with the animals throughout the study for example, blood samples are collected by laboratory personnel very frequently and the catheter placements require that some degree of physical contact be made in order to draw the samples from the surgically placed catheters. Animals must remain in the harness for the duration of the experiments due to need for

	continuous infusions of tracers and substrates used in each protocol. The harnesses are comfortable and the animals can either stand or relax in the harness. The room is approximately 300-350 sq ft.
Duration, frequency and interval of restraint	Up To 6 Hrs
Rationale for this type of restraint	These experiments address the effects of hormones and substrates on metabolic physiolgy. The studies require constant infusion of these compounds and samples are collected frequently (up to five minute intervals). There are multiple sampling sites and intravenous and arterial cannulas that require attention and care. Therefore maintaining the animals in a confined but comfortable postion is necessary.

Select the room where this procedure will be performed. If the room is not on the displayed list, you will need to go back to the PI Contact Information Page and add the room there and then return to select it for this procedure.

Select Room(s)	Med Center North/CC1343;;Med Center North/CC2323;;

XII d 9. Hazardous Agents

Identify any hazardous agents that will be used in the protocol in this section, and complete all fields. You will be contacted by Vanderbilt Environmental Health and Safety (VEHS) to develop and provide safe handling practices for your hazardous agents outside of the eSIRIUS process. If you select a previously approved APC from your list, VEHS may still need to contact you with additional questions or to verify information.

Species Name	Dog				
Agent	Type	Amount Administered	Route	Other Routes	Duration of Exposure of Animals
triated glucose	RAD	0.02uci/kg/min	IV		6 hr

XII d 9 i. Hazardous Agents Information

Species Name	Dog

Create an APC by filling out the following information completely. You will be contacted by Vanderbilt Environmental Health and Safety

(VEHS) to develop and provide safe handling practices for your hazardous agents, outside of the eSirius review process.

GAV=Gavage IC=Intracardiac ICRAN=Intracranial ID=Intradermal IM=Intramuscular
IMR=Immersion IN=Intranasal INH=Inhalation IP=Intraperitonial IT=Intrathecal
IV=Intravenous IVT=Intravitreal OTHER=Other PO=Orally SC=Subcutaneous
TOP=Topical

APC Name	3-H3 d-glucose
APC Number	R10038
Hazardous Agent	triated glucose
Category	RAD
Route of administration (check all applicable)	IV
If route is not on list, please select "Other" above and specify other route(s)	
Amount administered (dosage, volume and vehicle)	0.02uci/kg/min
Vehicle	normal saline (50-100 ml)
Duration of exposure of animals (frequency and interval)	6 hr

XII d 10. Drugs/Exogenous Substances (Non Hazardous)

Species Name	Dog

If you intend to use a neuromuscular blocker, please review the Use of Neuromuscular Blocking Drugs Policy. Click to view the guidelines on injection sites and volumes. Provide strong scientific justification for the use of NMBs. Describe all procedures involving the use of NMBs in sufficient detail, including anesthetic regimen, neuromuscular blocking drug(s) administered (generic name, dose, route of administration, duration of action, use of reversal agent, if applicable), timing of administration, and methods used to monitor animals during

the period of paralysis. Ensure appropriate training of personnel and documentation of procedures in accordance with relevant IACUC policies and guidelines.

Agent	Associated Procedures
Buprenorphine	
Glucagon	
Insulin	
Isoflurane	
Meloxicam	
Pentobarbital	
Propofol	
Somatostatin	
cefpodoxime proxetil	
cephalosporin	
d-Glucose	
heparin-glycerin	
indocyanine green	
lidocaine/bupivacaine	
normol saline	
polymeric glucose	

Drug/Substance Information

Species Name	Dog

Enter information pertaining to selected drug.

GAV=Gavage IC=Intracardiac ICRAN=IntracranialID=Intradermal IM=Intramuscular
IMR=Immersion IN=Intranasal INH=Inhalation IP=IntraperitonialIT=Intrathecal
IV=Intravenous IVT=IntravitrealOTHER=Other PO=Orally SC=Subcutaneous
TOP=Topical

Drug/Substance Name	Pentobarbital
Dose (mg/kg) Note: Investigators using rats and mice must adhere to VU "Guidelines on Maximum Administration Volumes by Route (https://www4.vanderbilt.edu/acup/iacuc/policies/sops/IACUC.SOP.AWEL.08_Max_Volumes.pdf)"	20-30 mg/kg (to effect)
Route of administration (check all applicable)	IV
Frequency of Administration	Once
Reason for Administration	general anesthesia
List all associated procedures/surgeries (only list the procedures; do not provide any detail here):	

Drug/Substance Information

Species Name	Dog

Enter information pertaining to selected drug.

GAV=Gavage IC=Intracardiac ICRAN=Intracranial ID=Intradermal IM=Intramuscular
IMR=Immersion IN=Intranasal INH=Inhalation IP=Intraperitonial IT=Intrathecal
IV=Intravenous IVT=Intravitreal OTHER=Other PO=Orally SC=Subcutaneous
TOP=Topical

Drug/Substance Name	Isoflurane
Dose (mg/kg) Note: Investigators using rats and mice must adhere to VU "Guidelines on Maximum Administration Volumes by Route (https://www4.vanderbilt.edu/acup/iacuc/policies/sops/IACUC.SOP.AWEL.08_Max_Volumes.pdf)"	To Effect--1-5% w O2
Route of administration (check all applicable)	INH
Frequency of Administration	Duration Of Procedure
Reason for Administration	general anesthesia
List all associated procedures/surgeries (only list the procedures; do not provide any detail here):	

Drug/Substance Information

Species Name	Dog

Enter information pertaining to selected drug.

GAV=Gavage IC=Intracardiac ICRAN=Intracranial ID=Intradermal IM=Intramuscular
IMR=Immersion IN=Intranasal INH=Inhalation IP=Intraperitonial IT=Intrathecal
IV=Intravenous IVT=Intravitreal OTHER=Other PO=Orally SC=Subcutaneous
TOP=Topical

Drug/Substance Name	Buprenorphine
Dose (mg/kg) Note: Investigators using rats and mice must adhere to VU "Guidelines on Maximum Administration Volumes by Route (https://www4.vanderbilt.edu/acup/iacuc/policies/sops/IACUC.SOP.AWEL.08_Max_Volumes.pdf)"	0.02 Mg/kg
Route of administration (check all applicable)	IM;SC
Frequency of Administration	30 Min Prior To All Surg.; 8 Hr Later For Laparotomy
Reason for Administration	Analgesia
List all associated procedures/surgeries (only list the procedures; do not provide any detail here):	

Drug/Substance Information

Species Name	Dog

Enter information pertaining to selected drug.

GAV=Gavage IC=Intracardiac ICRAN=IntracranialID=Intradermal IM=Intramuscular
IMR=Immersion IN=Intranasal INH=Inhalation IP=IntraperitonialIT=Intrathecal
IV=Intravenous IVT=IntravitrealOTHER=Other PO=Orally SC=Subcutaneous
TOP=Topical

Drug/Substance Name	Propofol
Dose (mg/kg) Note: Investigators using rats and mice must adhere to VU "Guidelines on Maximum Administration Volumes by Route (https://www4.vanderbilt.edu/acup/iacuc/policies/sops/IACUC.SOP.AWEL.08_Max_Volumes.pdf)"	4-6
Route of administration (check all applicable)	IV
Frequency of Administration	Once To Effect
Reason for Administration	anesthesia induction
List all associated procedures/surgeries (only list the procedures; do not provide any detail here):	

Drug/Substance Information

Species Name	Dog

Enter information pertaining to selected drug.

GAV=Gavage IC=Intracardiac ICRAN=IntracranialID=Intradermal IM=Intramuscular
IMR=Immersion IN=Intranasal INH=Inhalation IP=IntraperitonialIT=Intrathecal
IV=Intravenous IVT=IntravitrealOTHER=Other PO=Orally SC=Subcutaneous
TOP=Topical

Drug/Substance Name	Meloxicam
Dose (mg/kg) Note: Investigators using rats and mice must adhere to VU "Guidelines on Maximum Administration Volumes by Route (https://www4.vanderbilt.edu/acup/iacuc/policies/sops/IACUC.SOP.AWEL.08_Max_Volumes.pdf)"	0.2 On First Day, Followed By 0.1 Daily for one day
Route of administration (check all applicable)	PO;SC
Frequency of Administration	30 Min Prior To All Surg.; 24 Hr Later For Laparotomy
Reason for Administration	analgesia

List all associated procedures/surgeries (only list the procedures; do not provide any detail here):	

Drug/Substance Information

Species Name	Dog

Enter information pertaining to selected drug.

GAV=Gavage IC=Intracardiac ICRAN=IntracranialID=Intradermal IM=Intramuscular
IMR=Immersion IN=Intranasal INH=Inhalation IP=IntraperitonialIT=Intrathecal
IV=Intravenous IVT=IntravitrealOTHER=Other PO=Orally SC=Subcutaneous
TOP=Topical

Drug/Substance Name	Insulin
Dose (mg/kg) Note: Investigators using rats and mice must adhere to VU "Guidelines on Maximum Administration Volumes by Route (https://www4.vanderbilt.edu/acup/iacuc/policies/sops/IACUC.SOP.AWEL.08_Max_Volumes.pdf)"	0.10-5.0 uU/kg/min, 40-60 ml
Route of administration (check all applicable)	IV
Frequency of Administration	Continuously Throughout Experiment
Reason for Administration	pancreatic hormone replacement
List all associated procedures/surgeries (only list the procedures; do not provide any detail here):	

Drug/Substance Information

Species Name	Dog

Enter information pertaining to selected drug.

GAV=Gavage IC=Intracardiac ICRAN=IntracranialID=Intradermal IM=Intramuscular
IMR=Immersion IN=Intranasal INH=Inhalation IP=IntraperitonialIT=Intrathecal
IV=Intravenous IVT=IntravitrealOTHER=Other PO=Orally SC=Subcutaneous
TOP=Topical

Drug/Substance Name	indocyanine green
Dose (mg/kg) Note: Investigators using rats and mice must adhere to VU "Guidelines on Maximum Administration Volumes by Route (https://www4.vanderbilt.edu/acup/iacuc/policies/sops/IACUC.SOP.AWEL.08_Max_Volumes.pdf)"	0.10 Mg/m2/min
Route of administration (check all applicable)	IV
Frequency of Administration	Continuously Throughout Experiment

Reason for Administration	estimation of blood flow
List all associated procedures/surgeries (only list the procedures; do not provide any detail here):	

Drug/Substance Information

Species Name	Dog

Enter information pertaining to selected drug.

GAV=Gavage IC=Intracardiac ICRAN=Intracranial ID=Intradermal IM=Intramuscular
IMR=Immersion IN=Intranasal INH=Inhalation IP=Intraperitonial IT=Intrathecal
IV=Intravenous IVT=Intravitreal OTHER=Other PO=Orally SC=Subcutaneous
TOP=Topical

Drug/Substance Name	normol saline
Dose (mg/kg) Note: Investigators using rats and mice must adhere to VU "Guidelines on Maximum Administration Volumes by Route (https://www4.vanderbilt.edu/acup/iacuc/policies/sops/IACUC.SOP.AWEL.08_Max_Volumes.pdf)"	10-20 ml/kg/hr
Route of administration (check all applicable)	IV
Frequency of Administration	Continuously Throughout Surgery And Experiment
Reason for Administration	Fluid maintenance
List all associated procedures/surgeries (only list the procedures; do not provide any detail here):	

Drug/Substance Information

Species Name	Dog

Enter information pertaining to selected drug.

GAV=Gavage IC=Intracardiac ICRAN=Intracranial ID=Intradermal IM=Intramuscular
IMR=Immersion IN=Intranasal INH=Inhalation IP=Intraperitonial IT=Intrathecal
IV=Intravenous IVT=Intravitreal OTHER=Other PO=Orally SC=Subcutaneous
TOP=Topical

Drug/Substance Name	cephalosporin
Dose (mg/kg) Note: Investigators using rats and mice must adhere to VU "Guidelines on Maximum Administration Volumes by Route (https://www4.vanderbilt.edu/acup/iacuc/policies/sops/IACUC.SOP.AWEL.08_Max_Volumes.pdf)"	20
Route of administration (check all applicable)	SC
Frequency of Administration	Once Pre-operatively
Reason for Administration	antibiotic (cefazolin)
List all associated procedures/surgeries (only list the procedures; do not provide any detail here):	

Drug/Substance Information

Species Name	Dog

Enter information pertaining to selected drug.

GAV=Gavage IC=Intracardiac ICRAN=Intracranial ID=Intradermal IM=Intramuscular
IMR=Immersion IN=Intranasal INH=Inhalation IP=Intraperitonial IT=Intrathecal
IV=Intravenous IVT=Intravitreal OTHER=Other PO=Orally SC=Subcutaneous
TOP=Topical

Drug/Substance Name	cefpodoxime proxetil
Dose (mg/kg) Note: Investigators using rats and mice must adhere to VU "Guidelines on Maximum Administration Volumes by Route (https://www4.vanderbilt.edu/acup/iacuc/policies/sops/IACUC.SOP.AWEL.08_Max_Volumes.pdf)"	10
Route of administration (check all applicable)	PO
Frequency of Administration	Once Daily For 3 Days
Reason for Administration	antibiotic
List all associated procedures/surgeries (only list the procedures; do not provide any detail here):	

Drug/Substance Information

Species Name	Dog

Enter information pertaining to selected drug.

GAV=Gavage IC=Intracardiac ICRAN=Intracranial ID=Intradermal IM=Intramuscular
IMR=Immersion IN=Intranasal INH=Inhalation IP=Intraperitonial IT=Intrathecal
IV=Intravenous IVT=Intravitreal OTHER=Other PO=Orally SC=Subcutaneous
TOP=Topical

Drug/Substance Name	heparin-glycerin
Dose (mg/kg) Note: Investigators using rats and mice must adhere to VU "Guidelines on Maximum Administration Volumes by Route (https://www4.vanderbilt.edu/acup/iacuc/policies/sops/IACUC.SOP.AWEL.08_Max_Volumes.pdf)"	0.30 ml 1 (1000U/ml, heparin):1 (95% glycerin) Ratio
Route of administration (check all applicable)	IV
Frequency of Administration	Once
Reason for Administration	Filling solution for vascular catheter
List all associated procedures/surgeries (only list the procedures; do not provide any detail here):	

Drug/Substance Information

Species Name	Dog

Enter information pertaining to selected drug.

GAV=Gavage IC=Intracardiac ICRAN=IntracranialID=Intradermal IM=Intramuscular
IMR=Immersion IN=Intranasal INH=Inhalation IP=IntraperitonialIT=Intrathecal
IV=Intravenous IVT=IntravitrealOTHER=Other PO=Orally SC=Subcutaneous
TOP=Topical

Drug/Substance Name	Somatostatin
Dose (mg/kg) Note: Investigators using rats and mice must adhere to VU "Guidelines on Maximum Administration Volumes by Route (https://www4.vanderbilt.edu/acup/iacuc/policies/sops/IACUC.SOP.AWEL.08_Max_Volumes.pdf)"	0.8 ug/kgmin, 40-60 ml
Route of administration (check all applicable)	IV
Frequency of Administration	Continuously During Experiment
Reason for Administration	inhibition of endocrine pancreas
List all associated procedures/surgeries (only list the procedures; do not provide any detail here):	

Drug/Substance Information

Species Name	Dog

Enter information pertaining to selected drug.

GAV=Gavage IC=Intracardiac ICRAN=IntracranialID=Intradermal IM=Intramuscular
IMR=Immersion IN=Intranasal INH=Inhalation IP=IntraperitonialIT=Intrathecal
IV=Intravenous IVT=IntravitrealOTHER=Other PO=Orally SC=Subcutaneous
TOP=Topical

Drug/Substance Name	Glucagon
Dose (mg/kg) Note: Investigators using rats and mice must adhere to VU "Guidelines on Maximum Administration Volumes by Route (https://www4.vanderbilt.edu/acup/iacuc/policies/sops/IACUC.SOP.AWEL.08_Max_Volumes.pdf)"	0.5-5.0 ng/kg/min, 40-60 ml
Route of administration (check all applicable)	IV
Frequency of Administration	Continuously During Experiment
Reason for Administration	pancreatic hormone replacement
List all associated procedures/surgeries (only list the procedures; do not provide any detail here):	

Drug/Substance Information

Species Name	Dog

Enter information pertaining to selected drug.

GAV=Gavage IC=Intracardiac ICRAN=IntracranialID=Intradermal IM=Intramuscular
IMR=Immersion IN=Intranasal INH=Inhalation IP=IntraperitonialIT=Intrathecal
IV=Intravenous IVT=IntravitrealOTHER=Other PO=Orally SC=Subcutaneous
TOP=Topical

Drug/Substance Name	d-Glucose
Dose (mg/kg) Note: Investigators using rats and mice must adhere to VU "Guidelines on Maximum Administration Volumes by Route (https://www4.vanderbilt.edu/acup/iacuc/policies/sops/IACUC.SOP.AWEL.08_Max_Volumes.pdf)"	1-20 mg/kg, 10-150 ml
Route of administration (check all applicable)	IV
Frequency of Administration	Continuosly During Experiment
Reason for Administration	glycemic maintenance
List all associated procedures/surgeries (only list the procedures; do not provide any detail here):	

Drug/Substance Information

Species Name	Dog

Enter information pertaining to selected drug.

GAV=Gavage IC=Intracardiac ICRAN=IntracranialID=Intradermal IM=Intramuscular
IMR=Immersion IN=Intranasal INH=Inhalation IP=IntraperitonialIT=Intrathecal
IV=Intravenous IVT=IntravitrealOTHER=Other PO=Orally SC=Subcutaneous
TOP=Topical

Drug/Substance Name	polymeric glucose
Dose (mg/kg) Note: Investigators using rats and mice must adhere to VU "Guidelines on Maximum Administration Volumes by Route (https://www4.vanderbilt.edu/acup/iacuc/policies/sops/IACUC.SOP.AWEL.08_Max_Volumes.pdf)"	0.7-1.5 G/kg
Route of administration (check all applicable)	PO
Frequency of Administration	Once During Oral Glucose Tolerance Test
Reason for Administration	determination of glucose tolerance
List all associated procedures/surgeries (only list the procedures; do not provide any detail here):	

Drug/Substance Information

Species Name	Dog

Enter information pertaining to selected drug.

GAV=Gavage IC=Intracardiac ICRAN=IntracranialID=Intradermal IM=Intramuscular
IMR=Immersion IN=Intranasal INH=Inhalation IP=IntraperitonialIT=Intrathecal
IV=Intravenous IVT=IntravitrealOTHER=Other PO=Orally SC=Subcutaneous
TOP=Topical

Drug/Substance Name	lidocaine/bupivacaine
Dose (mg/kg) Note: Investigators using rats and mice must adhere to VU "Guidelines on Maximum Administration Volumes by Route (https://www4.vanderbilt.edu/acup/iacuc/policies/sops/IACUC.SOP.AWEL.08_Max_Volumes.pdf)"	4 mg/kg/1.5 mg/kg, Lido./bupiv. respectively, 3-4 ml, to effect
Route of administration (check all applicable)	SC
Frequency of Administration	Once
Reason for Administration	local anesthetic
List all associated procedures/surgeries (only list the procedures; do not provide any detail here):	

XII d 11. Euthanasia Methods

Species Name	Dog
Method	
Barbituate Overdose	

Euthanasia Method Information

Click link to view the American Veterinary Medical Association report on euthanasia AVMA Website - 2007 Euthanasia Guidelines. Click here to view Vanderbilt's policy on euthanasia.

Species Name	Dog

Choose Euthansia method. If desired method is not on list, please enter in **Other Euthanasia Method**.

Euthanasia Method	Barbituate Overdose
AVMA Classification	Acceptable

If method involves use of drugs, please specify drugs to be used and dosage for each.

IC=Intracardiac
IM=Intramuscular
INH=Inhalation
PO=Orally
ICRAN=IntracranialID=Intradermal
IMR=Immersion IN=Intranasal
IP=IntraperitonialIV=Intravenous
SC=Subcutaneous TOP=Topical

Agent	Dose	Administration Route
Pentobarbital [Analgesic/anesthetic]	125 Mg/kg	IV

If you are proposing to use a conditionally acceptable or unacceptable method for the species used, please provide a justification.

The use of CO2 to euthanize rodents should be subsequently followed by a secondary method to ensure death (e.g. cervical dislocation, decapitation or a thoracotomy). If using CO2 to euthanize rodents, indicate how you will verify death prior to carcass disposal.

The use of inhalation agents requires special consideration for scavenging waste gases and limiting exposure of personnel. If using an inhalation agent to euthanize animals, indicate how the waste gases will be scavenged (e.g. F/Air cannister, externally ducted hood).

Euthanasia location

Select the room where this procedure will be performed. If the room is not on the displayed list, you will need to go back to the PI Contact Information Page and add the room there and then return to select it for this procedure.

Select Room(s)	Med Center North/CC1343;;Med Center North/CC2323;;

XII d 13. USDA Pain Categories

Please enter the number of animals requested for each of the required categories. The total of the required fields may not exceed your total requested number.

If entering numbers for mouse (mus musculus) or rat (rattus rattus), determine the highest pain level relevant to your protocol and put all of your animals in that category. This will greatly simplify the process of tracking usage for rodents.

Click here to view the USDA Pain Categories. To reference Vanderbilt University's guidelines for determining USDA pain/distress levels, please click here.

For reference the USDA Categories are defined
E = unalleviated pain or distress
D = alleviated pain or distress
C = no pain or distress
B = animal held for breeding and/or not yet used in research

Species Name	Dog
Type	Procedure
NON SURGICAL	Blood/fluid/tissue Collection (ante-mortem)
NON SURGICAL	Diet Manipulation, Including Food/water Regulation
RESTRAINT	Pavlov's Harness
SURGICAL	Biopsy: Intra-abdominal/laporotomy
SURGICAL	Vascular catheter access
SURGICAL	Placement of catheters into subcutaneous space
SURGICAL	Laparotomy-Vascular Cannulations and Renal Sympathectomy
Total # Requested for Species	13

Pain Level	# of Animals	Description
D	13	Alleviated pain or distress

XII d 14. Adverse Consequences

Use this section to discuss all procedures or conditions that may be accompanied by pain, distress, or discomfort. Include discussion of infectious or spontaneous disease studies and transgenic animals, even if clinical signs or abnormal phenotypes are not expected.
Describe the monitoring plan for pain and distress, including frequency and duration of checking for health or behavioral abnormalities.

Species Name	Dog

Describe expected adverse consequences and severe complications that the animals may experience as a result of the procedures or test compounds described in this protocol. Examples of expected adverse consequences include loss of appetite, post-operative discomfort, pain at an injection site, or loss of animal life. Examples of severe complications include post-operative infections, wound dehiscence, hemorrhage, ventricular fibrillation, paralysis, anesthetic death, etc.
Surgical: As with any surgical procedure complications may arise. These include infection, vascular stasis, thrombosis and hemorrhage. Complications resulting from surgical intervention will be addressed immediately and appropriately depending on the existing complication. For infections, appropriate antibiotics, analgesics and/or anti-inflammatory compounds will be used. Vascular problems, thrombosis, physical impairment or hemorrhage will be treated as required including vascular repair and/or transfusion. Bilateral renal sympathectomy will not result in adverse consequences.<br><br>Experimental:glucose, insulin, glucagon and somatostatin will result in changes in the metabolic state of the animals. The changes can include hyperglycemia or hypoglycemia depending on the particular dose and protocol. The changes are within physiological ranges and do not result in pain or distress to the animals. They do however result in the way the body maintains glucose and utilizes substrates for energy requirements. The high fat,high carbohydrate dietary regimine administered over this period of time will result in mild insulin resistance however this level of insulin resistance does not manifest itself clinically with excessive hyperglycemia that would require diabetes therapy.
Monitoring Plan
Surgical: twice daily for the first three days

post-operativly for procedures involving laparotomy and daily thereafter until the conclusion of the terminal experiment.<br><br>Experimental: continuously throughout the experiment (up to 8 hr). Animals are in constant visual contact with laboratory personnel throughout the experiment. Assessments of concentrations of metabolic substrates, hematocrits, blood flow are performed and recorded at regular intervals (minimum at hourly intervals)throughout the experiment.
At what point will an animal be removed from the study or euthanized. How will this be determined?
Surgical:If other complications result in a deviation of pre-experimental criteria the animals will be euthanized. Overall humane endpoints could include but are not limited to anemia as evidenced by PCV of 25% or less, uncontrollable infection as evidenced by swelling, redness, tenderness, oozing from the incision site, fever, lethargy, vocalization, changed behavior (aggressiveness or retraction from social interactions depending on the demeanor of the particular animal), paralysis, diminished responsiveness or incoherence, self-trauma, and weight loss (loss of weight equal or greater than 20% and animals will be weighed at biweekly intervals.<br><br>Experimental: In any case where the metabolic experimental procedures results in pain or distress to the animals the compounds will be discontinued or the animal euthanized. Any demonstrated stress (dyspnea, weakness,) in addition to metabolic conditions mentioned above will result in termination of the procedure or early euthanasia of the animal.

XIII. Personnel List

Include all personnel on this project working with live animals or animal tissues. All individuals working with animals are required to complete relevant training. Refer to the AALAS Learning Library. All individuals working with animals or their tissues are required to satisfy occupational health requirements. Refer to the Vanderbilt Occupational Health Website for more information.

Click on the hyperlink in the "Name" column to edit information for that person. Note: The "Requester" field applies when someone other than the PI initiates a new protocol application. If the PI starts the protocol, "Requester" is blank or "No". Be sure to click on the PI’s name to answer all required questions.

Submit an Application for a New Protocol

Name	Phone	Email Id	Organization	Department	Pers Id	Primary Contact	Requestor	Employee ID
Cherrington, Alan D	(615) 322-7013	alan.cherrington@vanderbilt.edu	Vanderbilt University Medical Center	Molecular Physiology &Biophysics	11			
Williams, Phillip E	322-3639	phil.williams@vanderbilt.edu	Vanderbilt University Medical Center	Surgery - Surgical Research	266		Yes	
Moore, Mary C	(615) 343-0579	genie.moore@vanderbilt.edu	Vanderbilt University Medical Center	Molecular Physiology &Biophysics	32858			
Smith, Marta	322-7014	marta.smith@vanderbilt.edu	Vanderbilt University Medical Center	Molecular Physiology &Biophysics	34191			
Farmer, Benjamin N	322-7014	ben.farmer@vanderbilt.edu	Vanderbilt University Medical Center	Molecular Physiology &Biophysics	34329			
Farmer, Tiffany	(615) 322-7014	tiffany.farmer@vanderbilt.edu	Vanderbilt University Medical Center	Molecular Physiology &Biophysics	34330			
Scott, Melanie	322-7014	melanie.scott@vanderbilt.edu	Vanderbilt University Medical Center	Molecular Physiology &Biophysics	34340			
Edgerton, Dale	343-3193	dale.edgerton@vanderbilt.edu	Vanderbilt University Medical Center	Molecular Physiology &Biophysics	34441			
Hastings, Jon	343-2357	jon.hastings@vanderbilt.edu	Vanderbilt University Medical Center	Molecular Physiology &Biophysics	34525			
Adcock, Jamie	322-2879	jamie.adcock@vanderbilt.edu	Vanderbilt University Medical Center	Surgery - Surgical Research	36354			

Kraft, Guillaume	322-7014	guillaume.kraft@vanderbilt.edu	Vanderbilt University Medical Center	Molecular Physiology &Biophysics	36456			
Gregory, Justin M	615-322-7014	justin.m.gregory.1@Vanderbilt.Edu	Vanderbilt University Medical Center	Molecular Physiology &Biophysics	40260			
Fultz, Mary S	615-343-7161	mary.s.fultz@vanderbilt.edu	Vanderbilt University Medical Center	Surgery - Surgical Research	42816			

Be sure to click "O/H Validation" once you have added all of your personnel and resolve any Occupational Health concerns before submitting your protocol in order to expedite the review process.

XIII a Personnel Information

Please review and update contact information

Name	Cherrington, Alan D
Organization/Department	Vanderbilt University Medical Center / Molecular Physiology &Bi
Phone	(615) 322-7013
Email	alan.cherrington@vanderbilt.edu
Fax	(615) 343-0490
Emergency Phone	(615) 662-4123
Cell Phone	
Pager	
VUNet Id	CHERRIAD
Is this person a Vanderbilt student?	No
If so, is this student paid a wage by Vanderbilt?	
Is this person a visiting researcher?	No
If so, is this researcher compensated in any way by Vanderbilt?	
Employee ID	0000758

Submit an Application for a New Protocol

Please review and update degrees

Degrees
Ph.D.

Please review and update experience and qualifications

Experience and Qualification
More than 50 years working with dogs in research. Involved in the initiation of canine surgery and research program at Vanderbilt University. Internationally known authority on metabolism in the dog. All on-line training up-to-date.

Please enter all information pertinent to this person’s activities on the requested protocol.

Will person be handling animal products?	YES
If yes, enter description of animal products	blood , urine , tissue
Will this person be entering rooms where animals are housed or used (e.g., procedure rooms or laboratories/cores)?	YES
Will person be performing animal procedures?	YES
Will person be performing surgeries?	NO
Will person be handling animal restraints?	NO
Will person be handling controlled substances?	YES
Will person be handling/exposed to Hazardous agents?	YES
Will person be handling animal	NO

euthanasia?	

Select species handled by this person

Species Name
Dog

Please review and update address information

Address 1	710 Light Hall
Address 2	21st, Ave. S.
City	Nashville
State	TN
Zip	37232
Country	Usa

Occupational Health Information

Risk Category	

Personnel Protocol Related Activities

Person Activities > Bio Hazard

Species Name	Agent	Type
Dog	triated glucose	RAD

Person Activities > Procedures

Species Name	Procedure Name
Dog	Blood/fluid/tissue Collection (ante-mortem)
Dog	Diet Manipulation, Including Food/water Regulation

Training profile

Principal Investigator	Cherrington, Alan D
Protocol Application Number	M/15/147

Please review and update contact information

Name	Cherrington, Alan D
Organization/Department	Vanderbilt University Medical Center
Phone	(615) 322-7013
Email	alan.cherrington@vanderbilt.edu
Fax	(615) 343-0490
Emergency Phone	(615) 662-4123
Cell Phone	
Pager	
VUNet Id	CHERRIAD

Training Event	Type	Date Completed	Date Certified	Species?	Drugs?	Agents?	Euthansia?	Restraint?	Procedures?	Surgery?	Roles?	Date Enrolled
Ethics and Regulation in Animal Research	OAWA Training	06/16/2009	/ /	Yes								/ /
Introduction to Dogs	OAWA Training	09/01/2006	/ /	Yes								/ /
Working with the IACUC	OAWA Training	07/01/2002	/ /	Yes								/ /

XIII a Personnel Information

Please review and update contact information

Name	Williams, Phillip E
Organization/Department	Vanderbilt University Medical Center / Surgery - Surgical Resear
Phone	322-3639
Email	phil.williams@vanderbilt.edu
Fax	343-1355
Emergency Phone	322-2096
Cell Phone	615-516-4955
Pager	
VUNet Id	WILLIAPE
Is this person a Vanderbilt student?	No
If so, is this student paid a wage by Vanderbilt?	No
Is this person a visiting researcher?	No

If so, is this researcher compensated in any way by Vanderbilt?	No
Employee ID	0000862

Please review and update degrees

Degrees
B.S.

Please review and update experience and qualifications

Experience and Qualification
Protocol Related Activities: surgery, surgery assistance, anesthesia, euthanasia, husbandry.<br>Mr. Williams is a Research Associate Professor in Surgery and has 40 years experience in an academic research environment. Mr. Williams has 40 years experience with canine, porcine, ovine, caprine and rodent species, specifically with the surgical, anesthetic and experimental procedures relevant to this protocol. During this time he has provided direct supervision of research assistants, fellows, graduate students and medical students in surgical techniques (especially for chronic vascular cannulations), aseptic procedures, animal handling and non surgical procedures, immediate and chronic post- operative and pre-experimental animal care and care of the animal during the experiment.

Please enter all information pertinent to this person’s activities on the requested protocol.

Will person be handling animal products?	YES
If yes, enter description of animal products	blood, urine , tissue
Will this person be entering rooms where animals are housed or used (e.g., procedure rooms or laboratories/cores)?	YES
Will person be	YES

performing animal procedures?	
Will person be performing surgeries?	YES
Will person be handling animal restraints?	YES
Will person be handling controlled substances?	YES
Will person be handling/exposed to Hazardous agents?	YES
Will person be handling animal euthanasia?	YES

Select species handled by this person

Species Name
Dog

Please review and update address information

Address 1	
Address 2	
City	
State	
Zip	
Country	USA

Occupational Health Information

Risk Category	

Personnel Protocol Related Activities

Person Activities > Euthanasia

Species Name	Euthanasia Method
Dog	Barbituate Overdose

Person Activities > Restraint

Species Name	Restraint Type
Dog	Pavlov’s Harness

Person Activities > Surgeries

Species Name	Surgery Type
Dog	Biopsy: Intra-abdominal/laporotomy
Dog	Laparotomy-Vascular Cannulations and Renal Sympathectomy
Dog	Vascular catheter access

Person Activities > Bio Hazard

Species Name	Agent	Type
Dog	triated glucose	RAD

Person Activities > Procedures

Species Name	Procedure Name
Dog	Blood/fluid/tissue Collection (ante-mortem)
Dog	Diet Manipulation, Including Food/water Regulation

Training profile

Principal Investigator	Cherrington, Alan D
Protocol Application Number	M/15/147

Please review and update contact information

Name	Williams, Phillip E
Organization/Department	Vanderbilt University Medical Center
Phone	322-3639
Email	phil.williams@vanderbilt.edu
Fax	343-1355
Emergency Phone	322-2096
Cell Phone	615-516-4955
Pager	
VUNet Id	WILLIAPE

Training Event	Type	Date Completed	Date Certified	Species?	Drugs?	Agents?	Euthansia?	Restraint?	Procedures?	Surgery?	Roles?	Date Enrolled
Ethics and Regulation in Animal	OAWA Training	06/16/2009	/ /	Yes								/ /

Research												
Introduction to Dogs	OAWA Training	09/01/2006	/ /	Yes								/ /
Working with the IACUC	OAWA Training	04/01/2002	/ /	Yes								/ /

XIII a Personnel Information

Please review and update contact information

Name	Moore, Mary C
Organization/Department	Vanderbilt University Medical Center / Molecular Physiology &Bi
Phone	(615) 343-0579
Email	genie.moore@vanderbilt.edu
Fax	(615) 343-0490
Emergency Phone	(615)645-1640
Cell Phone	
Pager	
VUNet Id	MOOREMC
Is this person a Vanderbilt student?	No
If so, is this student paid a wage by Vanderbilt?	
Is this person a visiting researcher?	No
If so, is this researcher compensated in any way by Vanderbilt?	
Employee ID	0010371

Please review and update degrees

Degrees
Ph.D., R.D.

Please review and update experience and qualifications

Experience and Qualification
30 yrs experience in canine metabolic research. This includes experience in surgical and experimental settings described in

this application. Completed all required on-line training.

Please enter all information pertinent to this person’s activities on the requested protocol.

Will person be handling animal products?	YES
If yes, enter description of animal products	blood, urine, tissue
Will this person be entering rooms where animals are housed or used (e.g., procedure rooms or laboratories/cores)?	YES
Will person be performing animal procedures?	YES
Will person be performing surgeries?	NO
Will person be handling animal restraints?	YES
Will person be handling controlled substances?	YES
Will person be handling/exposed to Hazardous agents?	YES
Will person be handling animal euthanasia?	YES

Select species handled by this person

Species Name
Dog

Please review and update address information

Address 1	702 Light Hall
Address 2	
City	Nashville
State	TN
Zip	37232-0615
Country	Usa

Occupational Health Information

Risk Category	

Personnel Protocol Related Activities

Person Activities > Euthanasia

Species Name	Euthanasia Method
Dog	Barbituate Overdose

Person Activities > Restraint

Species Name	Restraint Type
Dog	Pavlov’s Harness

Person Activities > Bio Hazard

Species Name	Agent	Type
Dog	triated glucose	RAD

Person Activities > Procedures

Species Name	Procedure Name
Dog	Blood/fluid/tissue Collection (ante-mortem)
Dog	Diet Manipulation, Including Food/water Regulation

Training profile

Principal Investigator	Cherrington, Alan D
Protocol Application Number	M/15/147

Please review and update contact information

Name	Moore, Mary C
Organization/Department	Vanderbilt University Medical Center
Phone	(615) 343-0579
Email	genie.moore@vanderbilt.edu
Fax	(615) 343-0490
Emergency Phone	(615)645-1640
Cell Phone	
Pager	
VUNet Id	MOOREMC

Training Event	Type	Date Completed	Date Certified	Species?	Drugs?	Agents?	Euthansia?	Restraint?	Procedures?	Surgery?	Roles?	Date Enrolled
Ethics and Regulation in Animal Research	OAWA Training	06/11/2009	/ /	Yes								/ /
Introduction to Dogs	OAWA Training	09/01/2006	/ /	Yes								/ /
Working with the IACUC	OAWA Training	09/01/2006	/ /	Yes								/ /

XIII a Personnel Information

Please review and update contact information

Name	Smith, Marta
Organization/Department	Vanderbilt University Medical Center / Molecular Physiology &Bi
Phone	322-7014
Email	marta.smith@vanderbilt.edu
Fax	
Emergency Phone	615-287-3759
Cell Phone	
Pager	
VUNet Id	SMITHMS1
Is this person a Vanderbilt student?	No
If so, is this student paid a wage by Vanderbilt?	
Is this person a visiting researcher?	No

If so, is this researcher compensated in any way by Vanderbilt?	
Employee ID	0010112

Please review and update degrees

Degrees
BS, MS

Please review and update experience and qualifications

Experience and Qualification
20 yrs working with research dogs. all required on-line training completed. She has haen trained to collect blood feed and take care of animals post surgery and perform metabolic studies

Please enter all information pertinent to this person’s activities on the requested protocol.

Will person be handling animal products?	YES
If yes, enter description of animal products	blood, urine, tissue
Will this person be entering rooms where animals are housed or used (e.g., procedure rooms or laboratories/cores)?	YES
Will person be performing animal procedures?	YES
Will person be performing surgeries?	YES
Will person be handling animal restraints?	YES
Will person be handling controlled substances?	YES

Will person be handling/exposed to Hazardous agents?	YES
Will person be handling animal euthanasia?	YES

Select species handled by this person

Species Name
Dog

Please review and update address information

Address 1	21st Avenue &Garland Street
Address 2	
City	Nashville
State	TN
Zip	37232-
Country	Usa

Occupational Health Information

Risk Category	

Personnel Protocol Related Activities

Person Activities > Euthanasia

Species Name	Euthanasia Method
Dog	Barbituate Overdose

Person Activities > Restraint

Species Name	Restraint Type
Dog	Pavlov's Harness

Person Activities > Surgeries

Species Name	Surgery Type
Dog	Biopsy: Intra-abdominal/laporotomy
Dog	Laparotomy-Vascular Cannulations and Renal Sympathectomy
Dog	Vascular catheter access

Person Activities > Bio Hazard

Species Name	Agent	Type
Dog	triated glucose	RAD

Person Activities > Procedures

Species Name	Procedure Name
Dog	Blood/fluid/tissue Collection (ante-mortem)
Dog	Diet Manipulation, Including Food/water Regulation

Training profile

Principal Investigator	Cherrington, Alan D
Protocol Application Number	M/15/147

Please review and update contact information

Name	Smith, Marta
Organization/Department	Vanderbilt University Medical Center
Phone	322-7014
Email	marta.smith@vanderbilt.edu
Fax	
Emergency Phone	615-287-3759
Cell Phone	
Pager	
VUNet Id	SMITHMS1

Training Event	Type	Date Completed	Date Certified	Species?	Drugs?	Agents?	Euthansia?	Restraint?	Procedures?	Surgery?	Roles?	Date Enrolled
Ethics and Regulation in Animal Research	OAWA Training	06/11/2009	/ /	Yes								/ /
Introduction to Dogs	OAWA Training	01/01/2007	/ /	Yes								/ /
Working with the IACUC	OAWA Training	09/01/2006	/ /	Yes								/ /

XIII a Personnel Information

Please review and update contact information

Name	Farmer, Benjamin
Organization/Department	Vanderbilt University Medical Center / Molecular Physiology &Bi
Phone	322-7014
Email	ben.farmer@vanderbilt.edu
Fax	
Emergency Phone	403-4577
Cell Phone	
Pager	
VUNet Id	FARMERBN
Is this person a Vanderbilt student?	No
If so, is this student paid a wage by Vanderbilt?	
Is this person a visiting researcher?	No
If so, is this researcher compensated in any way by Vanderbilt?	
Employee ID	0026261

Please review and update degrees

Degrees
BS

Please review and update experience and qualifications

Experience and Qualification
Mr. Farmer has over 14 years experience with canine husbandry, surgery and experimental techniques that are described in this application. He has completed all institutional training

Please enter all information pertinent to this person’s activities on the requested protocol.

Submit an Application for a New Protocol

Will person be handling animal products?	YES
If yes, enter description of animal products	blood, urine, tissue
Will this person be entering rooms where animals are housed or used (e.g., procedure rooms or laboratories/cores)?	YES
Will person be performing animal procedures?	YES
Will person be performing surgeries?	YES
Will person be handling animal restraints?	YES
Will person be handling controlled substances?	YES
Will person be handling/exposed to Hazardous agents?	YES
Will person be handling animal euthanasia?	YES

Select species handled by this person

Species Name
Dog

Please review and update address information

Address 1	710 Light Hall
Address 2	21st Ave. South
City	Nashville
State	TN
Zip	37232
Country	Usa

Occupational Health Information

Risk Category	

Personnel Protocol Related Activities

Person Activities > Euthanasia

Species Name	Euthanasia Method
Dog	Barbituate Overdose

Person Activities > Restraint

Species Name	Restraint Type
Dog	Pavlov’s Harness

Person Activities > Surgeries

Species Name	Surgery Type
Dog	Biopsy: Intra-abdominal/laporotomy

Person Activities > Bio Hazard

Species Name	Agent	Type
Dog	triated glucose	RAD

Person Activities > Procedures

Species Name	Procedure Name
Dog	Blood/fluid/tissue Collection (ante-mortem)
Dog	Diet Manipulation, Including Food/water Regulation

Training profile

Principal Investigator	Cherrington, Alan D
Protocol Application Number	M/15/147

Please review and update contact information

Name	Farmer, Benjamin N
Organization/Department	Vanderbilt University Medical Center
Phone	322-7014
Email	ben.farmer@vanderbilt.edu
Fax	
Emergency Phone	403-4577
Cell Phone	
Pager	
VUNet Id	FARMERBN

Training Event	Type	Date Completed	Date Certified	Species?	Drugs?	Agents?	Euthansia?	Restraint?	Procedures?	Surgery?	Roles?	Date Enrolled
Ethics and Regulation in Animal Research	OAWA Training	06/11/2009	/ /	Yes								/ /
Introduction to Dogs	OAWA Training	09/01/2006	/ /	Yes								/ /
Working with the IACUC	OAWA Training	04/01/2005	/ /	Yes								/ /

XIII a Personnel Information

Please review and update contact information

Name	Farmer, Tiffany
Organization/Department	Vanderbilt University Medical Center / Molecular Physiology &Bi
Phone	(615) 322-7014
Email	tiffany.farmer@vanderbilt.edu
Fax	
Emergency Phone	403-4577
Cell Phone	
Pager	
VUNet Id	RODEWATD
Is this person a Vanderbilt student?	No
If so, is this student paid a wage by Vanderbilt?	
Is this person a visiting researcher?	No

If so, is this researcher compensated in any way by Vanderbilt?	
Employee ID	0051958

Please review and update degrees

Degrees
M.Sc.

Please review and update experience and qualifications

Experience and Qualification
12+ years experience with canine experimental and surgical procedures described in this application since 2002.

Please enter all information pertinent to this person’s activities on the requested protocol.

Will person be handling animal products?	YES
If yes, enter description of animal products	blood, urine, tissue
Will this person be entering rooms where animals are housed or used (e.g., procedure rooms or laboratories/cores)?	YES
Will person be performing animal procedures?	YES
Will person be performing surgeries?	YES
Will person be handling animal restraints?	YES
Will person be handling controlled substances?	YES
Will person be handling/exposed to	YES

Hazardous agents?	
Will person be handling animal euthanasia?	YES

Select species handled by this person

Species Name
Dog

Please review and update address information

Address 1	831a Light Hall
Address 2	
City	Nashville
State	TN
Zip	37232
Country	Usa

Occupational Health Information

Risk Category	

Personnel Protocol Related Activities

Person Activities > Euthanasia

Species Name	Euthanasia Method
Dog	Barbituate Overdose

Person Activities > Restraint

Species Name	Restraint Type
Dog	Pavlov’s Harness

Person Activities > Surgeries

Species Name	Surgery Type
Dog	Biopsy: Intra-abdominal/laporotomy
Dog	Laparotomy-Vascular Cannulations and Renal Sympathectomy
Dog	Vascular catheter access

Person Activities > Bio Hazard

Species Name	Agent	Type
Dog	triated glucose	RAD

Person Activities > Procedures

Species Name	Procedure Name
Dog	Blood/fluid/tissue Collection (ante-mortem)
Dog	Diet Manipulation, Including Food/water Regulation

Training profile

Principal Investigator	Cherrington, Alan D
Protocol Application Number	M/15/147

Please review and update contact information

Name	Farmer, Tiffany
Organization/Department	Vanderbilt University Medical Center
Phone	(615) 322-7014
Email	tiffany.farmer@vanderbilt.edu
Fax	
Emergency Phone	403-4577
Cell Phone	
Pager	
VUNet Id	RODEWATD

Training Event	Type	Date Completed	Date Certified	Species?	Drugs?	Agents?	Euthansia?	Restraint?	Procedures?	Surgery?	Roles?	Date Enrolled
Ethics and Regulation in Animal Research	OAWA Training	06/11/2009	/ /	Yes								/ /
Introduction to Dogs	OAWA Training	09/01/2006	/ /	Yes								/ /
Working with the IACUC	OAWA Training	09/01/2006	/ /	Yes								/ /

XIII a Personnel Information

Please review and update contact information

Name	Scott, Melanie
Organization/Department	Vanderbilt University Medical Center / Molecular Physiology &Bi
Phone	322-7014
Email	melanie.scott@vanderbilt.edu
Fax	
Emergency Phone	615-479-6179
Cell Phone	
Pager	
VUNet Id	SCOTTMF
Is this person a Vanderbilt student?	No
If so, is this student paid a wage by Vanderbilt?	
Is this person a visiting researcher?	No
If so, is this researcher compensated in any way by Vanderbilt?	
Employee ID	0006688

Please review and update degrees

Degrees
BS

Please review and update experience and qualifications

Experience and Qualification
35 years experience (13 veterinary + 17 research) to perform metabolic studies in dogs.<br>Completed all required on-line training.

Please enter all information pertinent to this person’s activities on the requested protocol.

Will person be handling animal products?	YES
If yes, enter description of animal products	blood, urine, tissue
Will this person be entering rooms where animals are housed or used (e.g., procedure rooms or laboratories/cores)?	YES
Will person be performing animal procedures?	YES
Will person be performing surgeries?	YES
Will person be handling animal restraints?	YES
Will person be handling controlled substances?	YES
Will person be handling/exposed to Hazardous agents?	YES
Will person be handling animal euthanasia?	YES

Select species handled by this person

Species Name
Dog

Please review and update address information

Address 1	710 Light Hall
Address 2	
City	Nashville
State	TN
Zip	37232
Country	Usa

Occupational Health Information

Risk Category	

Personnel Protocol Related Activities

Person Activities > Euthanasia

Species Name	Euthanasia Method
Dog	Barbituate Overdose

Person Activities > Restraint

Species Name	Restraint Type
Dog	Pavlov’s Harness

Person Activities > Surgeries

Species Name	Surgery Type
Dog	Biopsy: Intra-abdominal/laporotomy
Dog	Laparotomy-Vascular Cannulations and Renal Sympathectomy
Dog	Vascular catheter access

Person Activities > Bio Hazard

Species Name	Agent	Type
Dog	triated glucose	RAD

Person Activities > Procedures

Species Name	Procedure Name
Dog	Blood/fluid/tissue Collection (ante-mortem)
Dog	Diet Manipulation, Including Food/water Regulation

Training profile

Principal Investigator	Cherrington, Alan D
Protocol Application Number	M/15/147

Please review and update contact information

Name	Scott, Melanie
Organization/Department	Vanderbilt University Medical Center
Phone	322-7014
Email	melanie.scott@vanderbilt.edu
Fax	
Emergency Phone	615-479-6179
Cell Phone	
Pager	
VUNet Id	SCOTTMF

Training Event	Type	Date Completed	Date Certified	Species?	Drugs?	Agents?	Euthansia?	Restraint?	Procedures?	Surgery?	Roles?	Date Enrolled
Ethics and Regulation in Animal Research	OAWA Training	06/16/2009	/ /	Yes								/ /
Introduction to Dogs	OAWA Training	09/01/2006	/ /	Yes								/ /
Working with the IACUC	OAWA Training	07/01/2002	/ /	Yes								/ /

XIII a Personnel Information

Please review and update contact information

Name	Edgerton, Dale
Organization/Department	Vanderbilt University Medical Center / Molecular Physiology &Bi
Phone	343-3193
Email	dale.edgerton@vanderbilt.edu
Fax	
Emergency Phone	438-4918
Cell Phone	
Pager	
VUNet Id	EDGERTDS
Is this person a Vanderbilt student?	No
If so, is this student paid a wage by Vanderbilt?	
Is this person a visiting researcher?	No

If so, is this researcher compensated in any way by Vanderbilt?	
Employee ID	0033502

Please review and update degrees

Degrees
Ph.D.

Please review and update experience and qualifications

Experience and Qualification
20 yrs experience in canine metabolic research. This includes experience in surgical and experimental settings described in this application. completed all on-line training.

Please enter all information pertinent to this person's activities on the requested protocol.

Will person be handling animal products?	YES
If yes, enter description of animal products	blood, urine, tissue
Will this person be entering rooms where animals are housed or used (e.g., procedure rooms or laboratories/cores)?	YES
Will person be performing animal procedures?	YES
Will person be performing surgeries?	NO
Will person be handling animal restraints?	YES
Will person be handling controlled substances?	YES
	YES

Will person be handling/exposed to Hazardous agents?	
Will person be handling animal euthanasia?	YES

Select species handled by this person

Species Name
Dog

Please review and update address information

Address 1	21st Avenue &Garland Street
Address 2	
City	Nashville
State	TN
Zip	37232-
Country	Usa

Occupational Health Information

Risk Category	

Personnel Protocol Related Activities

Person Activities > Euthanasia

Species Name	Euthanasia Method
Dog	Barbituate Overdose

Person Activities > Restraint

Species Name	Restraint Type
Dog	Pavlov’s Harness

Person Activities > Bio Hazard

Species Name	Agent	Type
Dog	triated glucose	RAD

Person Activities > Procedures

Species Name	Procedure Name
Dog	Blood/fluid/tissue Collection (ante-mortem)
Dog	Diet Manipulation, Including Food/water Regulation

Training profile

Principal Investigator	Cherrington, Alan D
Protocol Application Number	M/15/147

Please review and update contact information

Name	Edgerton, Dale
Organization/Department	Vanderbilt University Medical Center
Phone	343-3193
Email	dale.edgerton@vanderbilt.edu
Fax	
Emergency Phone	438-4918
Cell Phone	
Pager	
VUNet Id	EDGERTDS

Training Event	Type	Date Completed	Date Certified	Species?	Drugs?	Agents?	Euthansia?	Restraint?	Procedures?	Surgery?	Roles?	Date Enrolled
Ethics and Regulation in Animal Research	OAWA Training	05/12/2009	/ /	Yes								/ /
Introduction to Dogs	OAWA Training	09/01/2006	/ /	Yes								/ /
Working with the IACUC	OAWA Training	05/01/2006	/ /	Yes								/ /

XIII a Personnel Information

Please review and update contact information

Name	Hastings, Jon
Organization/Department	Vanderbilt University Medical Center / Molecular Physiology &Bi
Phone	343-2357
Email	jon.hastings@vanderbilt.edu
Fax	
Emergency Phone	333-2757
Cell Phone	
Pager	

Submit an Application for a New Protocol

VUNet Id	HASTINGJ
Is this person a Vanderbilt student?	No
If so, is this student paid a wage by Vanderbilt?	
Is this person a visiting researcher?	No
If so, is this researcher compensated in any way by Vanderbilt?	Yes
Employee ID	0001698

Please review and update degrees

Degrees
BS

Please review and update experience and qualifications

Experience and Qualification
30 yrs experience experience in canine metabolic research. This includes experience in the experimental settings described in this application. All on-line training completed.

Please enter all information pertinent to this person's activities on the requested protocol.

Will person be handling animal products?	YES
If yes, enter description of animal products	blood, urine, tissue
Will this person be entering rooms where animals are housed or used (e.g., procedure rooms or laboratories/cores)?	YES
Will person be performing animal procedures?	YES

Will person be performing surgeries?	NO
Will person be handling animal restraints?	NO
Will person be handling controlled substances?	YES
Will person be handling/exposed to Hazardous agents?	YES
Will person be handling animal euthanasia?	NO

Select species handled by this person

Species Name
Dog

Please review and update address information

Address 1	21st Avenue &Garland Street
Address 2	
City	Nashville
State	TN
Zip	37232
Country	Usa

Occupational Health Information

Risk Category	

Personnel Protocol Related Activities

Person Activities > Bio Hazard

Species Name	Agent	Type
Dog	triated glucose	RAD

Person Activities > Procedures

Species Name	Procedure Name
Dog	Blood/fluid/tissue Collection (ante-mortem)
Dog	Diet Manipulation, Including Food/water Regulation

Training profile

Principal Investigator	Cherrington, Alan D
Protocol Application Number	M/15/147

Please review and update contact information

Name	Hastings, Jon
Organization/Department	Vanderbilt University Medical Center
Phone	343-2357
Email	jon.hastings@vanderbilt.edu
Fax	
Emergency Phone	333-2757
Cell Phone	
Pager	
VUNet Id	HASTINGJ

Training Event	Type	Date Completed	Date Certified	Species?	Drugs?	Agents?	Euthansia?	Restraint?	Procedures?	Surgery?	Roles?	Date Enrolled
Ethics and Regulation in Animal Research	OAWA Training	06/16/2009	/ /	Yes								/ /
Introduction to Dogs	OAWA Training	09/01/2006	/ /	Yes								/ /
Working with the IACUC	OAWA Training	09/01/2006	/ /	Yes								/ /

XIII a Personnel Information

Please review and update contact information

Name	Adcock, Jamie
Organization/Department	Vanderbilt University Medical Center / Surgery - Surgical Resear
Phone	322-2879
Email	jamie.adcock@vanderbilt.edu
Fax	
Emergency Phone	724-8452
Cell Phone	
Pager	

Submit an Application for a New Protocol

VUNet Id	YATESJM1
Is this person a Vanderbilt student?	NO
If so, is this student paid a wage by Vanderbilt?	NO
Is this person a visiting researcher?	NO
If so, is this researcher compensated in any way by Vanderbilt?	NO
Employee ID	0044598

Please review and update degrees

Degrees
LVMT, AAS

Please review and update experience and qualifications

Experience and Qualification
AALAS Online Training in canines. 20 years veterinary/ 15 years research experience. 2002 AAS in Applied Science, Veterinary Technology. credentialed in TN/ Mandatory 12 hr. CE annually. VA online courses completed.<br>Was trained in blood sampling, surgical procedures, restraint and pain management for multiple species including rodents. Protocol related activities include anesthesia, surgery, and euthanasia.

Please enter all information pertinent to this person's activities on the requested protocol.

Will person be handling animal products?	YES
If yes, enter description of animal products	blood, urine, tissue
Will this person be entering rooms where animals are housed or used (e.g., procedure rooms or	YES

laboratories/cores)?	
Will person be performing animal procedures?	YES
Will person be performing surgeries?	NO
Will person be handling animal restraints?	YES
Will person be handling controlled substances?	YES
Will person be handling/exposed to Hazardous agents?	YES
Will person be handling animal euthanasia?	YES

Select species handled by this person

Species Name
Dog

Please review and update address information

Address 1	Cc-2326mcn
Address 2	21st Ave. South
City	Nashville
State	TN
Zip	37232
Country	Usa

Occupational Health Information

Risk Category	

Personnel Protocol Related Activities

Person Activities > Euthanasia

Species Name	Euthanasia Method
Dog	Barbituate Overdose

Person Activities > Restraint

Species Name	Restraint Type
Dog	Pavlov’s Harness

Person Activities > Bio Hazard

Species Name	Agent	Type
Dog	triated glucose	RAD

Person Activities > Procedures

Species Name	Procedure Name
Dog	Blood/fluid/tissue Collection (ante-mortem)
Dog	Diet Manipulation, Including Food/water Regulation

Training profile

Principal Investigator	Cherrington, Alan D
Protocol Application Number	M/15/147

Please review and update contact information

Name	Adcock, Jamie
Organization/Department	Vanderbilt University Medical Center
Phone	322-2879
Email	jamie.adcock@vanderbilt.edu
Fax	
Emergency Phone	724-8452
Cell Phone	
Pager	
VUNet Id	YATESJM1

Training Event	Type	Date Completed	Date Certified	Species?	Drugs?	Agents?	Euthansia?	Restraint?	Procedures?	Surgery?	Roles?	Date Enrolled
Ethics and Regulation	OAWA Training	06/16/2009	/ /	Yes								/ /

in Animal Research												
Introduction to Dogs	OAWA Training	09/01/2006	/ /	Yes								/ /
Working with the IACUC	OAWA Training	01/01/2002	/ /	Yes								/ /

XIII a Personnel Information

Please review and update contact information

Name	Kraft, Guillaume
Organization/Department	Vanderbilt University Medical Center / Molecular Physiology &Bi
Phone	322-7014
Email	guillaume.kraft@vanderbilt.edu
Fax	343-0490
Emergency Phone	554-8976
Cell Phone	
Pager	
VUNet Id	KRAFTG
Is this person a Vanderbilt student?	No
If so, is this student paid a wage by Vanderbilt?	
Is this person a visiting researcher?	No
If so, is this researcher compensated in any way by Vanderbilt?	
Employee ID	0086772

Please review and update degrees

Degrees
PHD

Please review and update experience and qualifications

Experience and Qualification

9 years experience working with canines in this setting. He was trained by staff in surgical research who are manage the canine surgical and experimental facility.

Please enter all information pertinent to this person’s activities on the requested protocol.

Will person be handling animal products?	YES
If yes, enter description of animal products	food ; surgery &study items
Will this person be entering rooms where animals are housed or used (e.g., procedure rooms or laboratories/cores)?	YES
Will person be performing animal procedures?	NO
Will person be performing surgeries?	NO
Will person be handling animal restraints?	NO
Will person be handling controlled substances?	NO
Will person be handling/exposed to Hazardous agents?	NO
Will person be handling animal euthanasia?	NO

Select species handled by this person

Species Name
Dog

Please review and update address information

Address 1	2200 Pierce Ave , 710 Rrb
Address 2	
City	Nashville
State	TN
Zip	37232
Country	Usa

Occupational Health Information

Risk Category	

Personnel Protocol Related Activities

Training profile

Principal Investigator	Cherrington, Alan D
Protocol Application Number	M/15/147

Please review and update contact information

Name	Kraft, Guillaume
Organization/Department	Vanderbilt University Medical Center
Phone	322-7014
Email	guillaume.kraft@vanderbilt.edu
Fax	343-0490
Emergency Phone	554-8976
Cell Phone	
Pager	
VUNet Id	KRAFTG

Training Event	Type	Date Completed	Date Certified	Species?	Drugs?	Agents?	Euthansia?	Restraint?	Procedures?	Surgery?	Roles?	Date Enrolled
Ethics and Regulation in Animal Research	OAWA Training	06/16/2009	/ /	Yes								/ /
Introduction to Dogs	OAWA Training	01/28/2009	/ /	Yes								/ /
Working with the IACUC	OAWA Training	01/27/2009	/ /	Yes								/ /

XIII a Personnel Information

Please review and update contact information

Name	Gregory, Justin M
Organization/Department	Vanderbilt University Medical Center / Molecular Physiology &Bi
Phone	615-322-7014
Email	justin.m.gregory.1@Vanderbilt.Edu
Fax	615-343-0490
Emergency Phone	615-322-7002
Cell Phone	865-385-7673
Pager	
VUNet Id	GREGORJ2
Is this person a Vanderbilt student?	No
If so, is this student paid a wage by Vanderbilt?	
Is this person a visiting researcher?	No
If so, is this researcher compensated in any way by Vanderbilt?	
Employee ID	0100567

Please review and update degrees

Degrees
BS, MD

Please review and update experience and qualifications

Experience and Qualification
Dr. Gregory is a board certified endocrinologist and has 4 years experience working with canines. All institutional training requirements have been met.

Please enter all information pertinent to this person’s activities on the requested protocol.

Will person be handling animal products?	YES
If yes, enter description of animal products	SAMPLES(tissue, plasma, blood)
Will this person be entering rooms where animals are housed or used (e.g., procedure rooms or laboratories/cores)?	YES
Will person be performing animal procedures?	YES
Will person be performing surgeries?	NO
Will person be handling animal restraints?	YES
Will person be handling controlled substances?	NO
Will person be handling/exposed to Hazardous agents?	YES
Will person be handling animal euthanasia?	NO

Select species handled by this person

Species Name
Dog

Please review and update address information

Address 1	248 Westchase Dr.
Address 2	
City	Nashville
State	TN
Zip	37205
Country	Usa

Occupational Health Information

Risk Category	

Personnel Protocol Related Activities

Person Activities > Restraint

Species Name	Restraint Type
Dog	Pavlov’s Harness

Person Activities > Bio Hazard

Species Name	Agent	Type
Dog	triated glucose	RAD

Person Activities > Procedures

Species Name	Procedure Name
Dog	Blood/fluid/tissue Collection (ante-mortem)
Dog	Diet Manipulation, Including Food/water Regulation

Training profile

Principal Investigator	Cherrington, Alan D
Protocol Application Number	M/15/147

Please review and update contact information

Name	Gregory, Justin M
Organization/Department	Vanderbilt University Medical Center
Phone	615-322-7014
Email	justin.m.gregory.1@Vanderbilt.Edu
Fax	615-343-0490
Emergency Phone	615-322-7002
Cell Phone	865-385-7673
Pager	
VUNet Id	GREGORJ2

Training Event	Type	Date Completed	Date Certified	Species?	Drugs?	Agents?	Euthansia?	Restraint?	Procedures?	Surgery?	Roles?	Date Enrolled
Ethics and Regulation in Animal Research		/ /	/ /									/ /

Introduction to Dogs		/ /	/ /									/ /
Working with the IACUC		/ /	/ /									/ /

XIII a Personnel Information

Please review and update contact information

Name	Fultz, Mary S
Organization/Department	Vanderbilt University Medical Center / Surgery - Surgical Resear
Phone	615-343-7161
Email	mary.s.fultz@vanderbilt.edu
Fax	
Emergency Phone	615-574-0600
Cell Phone	615-574-0600
Pager	
VUNet Id	FULTZMS
Is this person a Vanderbilt student?	NO
If so, is this student paid a wage by Vanderbilt?	
Is this person a visiting researcher?	NO
If so, is this researcher compensated in any way by Vanderbilt?	
Employee ID	0139836

Please review and update degrees

Degrees
AD,LVT

Please review and update experience and qualifications

Experience and Qualification
Ms. Fultz has 16 years experience as a licensed veterinary technician in clinical veterinary practice. Her clinical veterinary experience is related to companion animals and

farm species including porcine and ovine species in clinical veterinary practice. Ms. Fultz will work under direct supervision of Mr. Williams and Ms. Adcock. Ms. Fultz has completed all required institutional training.

Please enter all information pertinent to this person's activities on the requested protocol.

Will person be handling animal products?	YES
If yes, enter description of animal products	blood, urine, tissue
Will this person be entering rooms where animals are housed or used (e.g., procedure rooms or laboratories/cores)?	YES
Will person be performing animal procedures?	YES
Will person be performing surgeries?	NO
Will person be handling animal restraints?	YES
Will person be handling controlled substances?	YES
Will person be handling/exposed to Hazardous agents?	YES
Will person be handling animal euthanasia?	YES

Select species handled by this person

Species Name
Dog

Please review and update address information

Address 1	Cc-2332 Mcn
Address 2	21st Ave. S.
City	Nashville
State	TN
Zip	37232
Country	Usa

Occupational Health Information

Risk Category	

Personnel Protocol Related Activities

Person Activities > Euthanasia

Species Name	Euthanasia Method
Dog	Barbituate Overdose

Person Activities > Restraint

Species Name	Restraint Type
Dog	Pavlov’s Harness

Person Activities > Bio Hazard

Species Name	Agent	Type
Dog	triated glucose	RAD

Person Activities > Procedures

Species Name	Procedure Name
Dog	Blood/fluid/tissue Collection (ante-mortem)
Dog	Diet Manipulation, Including Food/water Regulation

Training profile

Principal Investigator	Cherrington, Alan D
Protocol Application Number	M/15/147

Please review and update contact information

Name	Fultz, Mary S
Organization/Department	Vanderbilt University Medical Center
Phone	615-343-7161
Email	mary.s.fultz@vanderbilt.edu
Fax	
Emergency Phone	615-574-0600
Cell Phone	615-574-0600
Pager	
VUNet Id	FULTZMS

Training Event	Type	Date Completed	Date Certified	Species?	Drugs?	Agents?	Euthansia?	Restraint?	Procedures?	Surgery?	Roles?	Date Enrolled
Ethics and Regulation in Animal Research		/ /	/ /									/ /
Introduction to Dogs		/ /	/ /									/ /
Working with the IACUC		/ /	/ /									/ /

XIV. Databases Searched

For All Species

Experiments should be designed to avoid or minimize discomfort, distress and pain without compromising research goals using the so-called "3 Rs". The 3 Rs include: **replacement** with non-animal systems when possible, **reduction** in the number of animals required to obtain scientifically valid data through better experimental design and **refinement** techniques that decrease or eliminate pain or distress. Please consider "**refinement**" in the Search Results box below, as "**reduction**" and "**replacement**" should have been covered in other sections (Animal Number Justification and Justification for Animal Use and Choice of Species respectively) in this protocol.

The IACUC no longer requires a literature search for non-USDA Covered Species.

However, if you have not addressed "**refinement**" in this protocol, please do so in the Search Results box below.

The Database Search sections below must be complete if this is: 1) a training or teaching protocol, or 2) the reviewer(s) have asked you to address a specific

experimental procedure through a literature search.

For USDA Covered Species

The IACUC requires that experimental procedures avoid or minimize discomfort, distress and pain, see The United States Animal Welfare Act (AWA) regulations. Principal investigators are therefore required to consider alternatives to procedures that may cause more than momentary or slight pain or distress to animals. If your protocol involves more than momentary discomfort, distress and pain, you are required to perform a literature search for alternatives to these painful procedures using at least two literature databases. Otherwise, you can leave the database search blank.

For a list of commonly used literature databases to identify alternatives to painful procedures, click here.

For a partial (non-inclusive) list of keywords that are associated with commonly-employed painful procedures, click here.

For detailed information on how to perform a literature search for alternatives to painful procedures, please click here to reference the IACUC manual; please click non-human primates for a specific example.

Click here to view USDA/APHIS Policy #11 *Painful and Distressful Procedures*.

Click here to view USDA/APHIS Policy #12 *Consideration of Alternatives to Painful/Distressful Procedures*.

Question Updated: 12/10/2014

1st Database Searched
Medline
2nd Database Searched
Alternatives to Animal Testing on the Web (Altweb)
Keywords and search strategy used
in vivo model systems, animal pain alternatives, canine, surgery,laparotomy, vascular cannulations, hepatic, portal, renal, veins, femoral artery, femoral vein, renal sympathectomy, bilateral renal sympathectomy, kidney, renal, sympathetic nerve, liver, glucose uptake, glucose metabolism, hyperinsulinemia, hyperglycemia, euglycemnia, hypoglycemia, biopsies, subcutaneous catheter placement, pavlov harness, restraint, fasting, single housing
Search Results

The procedures outlined in this application require surgery and animals are studied while sitting in a pavlov harness. All animals will have a laparotomy for the purpose of vascular cannulations (hepatic and portal veins) and devices will be placed on vessels for the assessment of blood flow and femoral artery cannula. In addtion some of the animals will have the sympathetic nerves supplying the kidney transected to determine if thier role in the developmet of insulin resistance. The cannula and flow devices permit the evaluation of hepatic and whole body metabolism. Following a two week recovery, a metabolic experiment is conducted and the previously placed surgical devices are used to collect blood and measure blood flow while the animal sits in a pavlov harness. Reduction, refinement and replacement considerations have been thoroughly assessed and included in our literature search. Experimental model systems using tissue culture, cellular experimental systems and or/in situ perfusions would not address the complex neural and intraorgan relationships that exist and play a significant role in metabolic regulation. The numbers of animals that will be utilized with in these procedures are the minimal number required to generate a scientifically justifiable result. Finally, the impact of these procedures on potential pain and distress have been carefully considered and thoroughly reviewed by ourselves, veterinarians specializing in laboratory animal medicine, faculty both at this institution and at other institutions and thru careful review of the information and expertise provided by organizations associated with animal welfare. Our years of expertise and access to and assistance of veterinary staff have led us to develop effective analgesic regimens and obviate the potential painful or distressful procedures. Thorough literature searches were performed addressing in vivo model systems, animal pain alternatives, canine, surgery,laparotomy, vascular cannulations, hepatic, portal, renal, veins, femoral artery, femoral vein, renal sympathectomy, bilateral renal sympathectomy, kidney, renal, sympathetic nerve, liver, glucose uptake, glucose metabolism, hyperinsulinemia, hyperglycemia, euglycemnia, hypoglycemia, biopsies, subcutaneous catheter placement, pavlov harness, restraint, fasting, single housing
The searches were conducted utilizing several different combinations or sets of the above words. In addition

combinations of the word sets were also searched. 6 different word set combinations were searched, each set with a primary theme such as: vascular cannulations and laparotomy: in vivo and canine: liver glucose metabolism, etc. For example, to identify alternatives for the use of canines in the study of hepatic sympathectomy we utilized combinations of canine, alternatives, models, liver, sympathectomey, glucose. These searches on were conducted in Medline (1966-present) and Altweb on May 24, 2016. The citation yield of the searches varied depending on the word sets and when we combined the word sets the yield narrowed significantly. In all combination yields we reviewed the citations and did not find any alternative to the procedures that might result in pain or distress.
Consultation with colleagues (include name, qualification, etc.)
Journals used in search
Scientific meetings (seminars, focus groups, etc.)
Indicate whether alternatives were found and if so justify why they cannot be used.
No alternatives to the proposed procedures were identified. A metabolic experiment is conducted in the conscious animal and the previously placed surgical devices are used to collect blood and measure blood flow while the animal sits in a pavlov harness. The metabolic experiments cannot be conducted in the anesthetized state due to the significant metabolic effects of general anesthetics.

Inclusive Dates - From:	01/01/1966
To:	05/24/2016

Copyright ' 2002-2005, NTM Consulting Services, Inc.
3550 Mowry Avenue, Suite 201
Fremont, CA 94538

Submit an Application for a New Protocol

Phone: 510-744-3901 Fax: 510-744-3904
www.ntmcs.com
